# Prospective Memory Training in Older Adults: A Systematic Review and Meta-Analysis

**DOI:** 10.1007/s11065-022-09536-5

**Published:** 2022-05-11

**Authors:** Zita C. K. Tse, Yuan Cao, James M. Ogilvie, Bolton K. H. Chau, Daphne H. C. Ng, David H. K. Shum

**Affiliations:** 1 Department of Rehabilitation Sciences, The Hong Kong Polytechnic University, Hong Kong, Hong Kong; 2 Grififth Criminology Institute, Griffith University, Brisbane, Australia; 3 University Research Facility in Behavioral and Systems Neuroscience, The Hong Kong Polytechnic University, Hong Kong, Hong Kong; 4 Research Institute for Smart Ageing, The Hong Kong Polytechnic University, Hong Kong, Hong Kong; 5 Mental Health Research Centre, The Hong Kong Polytechnic University, Hong Kong, Hong Kong

**Keywords:** Prospective memory, Older adults, Meta-analysis, Ageing

## Abstract

Prospective memory (PM), which enables one to remember to carry out delayed intentions, is crucial for everyday functioning. PM commonly deteriorates upon cognitive decline in older adults, but several studies have shown that PM in older adults can be improved by training. The current study aimed to summarise this evidence by conducting a qualitative systematic analysis and quantitative meta-analysis of the effects of PM training in older adults, for which systematic searches were conducted across seven databases (Cochrane Library, Embase, PubMed, PsycInfo, Web of Science, CINAHL and Scopus). Forty-eight studies were included in the qualitative analysis, and 43% of the assessed PM training interventions showed positive gains in enhancing PM. However, the methodological quality varied across the studies, with 41% of the non-randomised control trials (non-RCTs) rated as having either serious or critical risk of bias. Therefore, only 29 RCTs were included in the subsequent quantitative meta-analysis. We found a significant and moderate immediate efficacy (Hedges’ *g* = 0.54) of PM training in enhancing PM performance in older adults, but no significant long-term efficacy (Hedges’ *g* = 0.20). Two subgroup analyses also revealed a robust training efficacy across the study population (i.e., healthy and clinical population) and the number of training sessions (i.e., single session and programme-based). Overall, this study provided positive evidence to support PM training in older adults. Further studies are warranted to explore the mechanisms by which PM training exerts its effects, and better-quality RCTs are needed to provide more robust evidence supporting our findings.

**Supplementary information:**

The online version contains supplementary material available at 10.1007/s11065-022-09536-5.

## Introduction

The global population of people aged over 65 years will increase dramatically between 2019 and 2050 and is estimated to reach approximately 1.5 billion by 2050 (United Nations, 2019). It is also anticipated this increase will amplify the medical and financial load on long-term healthcare provisions (Norton, 2016). Memory decline is one of the most common age-related cognitive impairments reported by older adults (Buckner, 2004). For example, age-related memory decline has been commonly reported by studies investigating retrospective memory (Sliwinski et al., 2003), episodic memory (Van Petten, 2004) and spatial memory (Erickson et al., 2010). The rapid growth of the older population and corresponding societal burden necessitates the identification of preventive measures and interventions that can ameliorate age-related cognitive decline.

Prospective memory (PM), which enables one to remember to carry out delayed intentions (McDaniel & Einstein, 2007; Shum & Fleming, 2018; Shum et al., 2017), deteriorates in older adults (Smith et al., 2000). Older adults often report a decline in the two common types of PM, namely event-based prospective memory (EBPM) and time-based prospective memory (TBPM; Cornelis et al., 2019; Henry et al., 2004; Park et al., 1997). To explain age-related PM decline in healthy older adults, the multiprocess theory postulates that PM failures could be attributed to difficulties in strategic monitoring processes during PM retrieval (Mullet et al., 2013) or in the disengagement and preparatory re-engagement of strategic monitoring (Ball et al., 2019). The preparatory attentional processes theory postulates that non-automatic resources are required to perform PM tasks (Smith et al., 2007). Thus, another possible explanation for age-related PM decline is the overall decline of cognitive resources in older adults. Not only have PM impairments been shown in terms of behaviour, but neural evidence has linked them to changes in the brain activities of older adults. Studies on the neural basis of this decline have reported that older adults exhibit a reduction in brain activity in the anterior prefrontal cortex and the dorsal frontoparietal cognitive control network when performing a PM task (Lamichhane et al., 2018). More importantly, older adults recruit additional brain areas such as the bilateral supplementary motor area, left inferior and right middle frontal cortices, and superior parietal and occipital cortices during EBPM tasks (Gonneaud et al., 2017), suggesting an age-related compensation of brain resources used for PM tasks.

In addition, older adults with clinical disorders appear to have poorer PM than healthy older adults. Previous studies have shown that PM is impaired in people with mild cognitive impairment (MCI; Schmitter-Edgecombe et al., 2009; Tam & Schmitter-Edgecombe, 2013), dementia (Maylor, 1995), stroke (Hogan et al., 2020) and Parkinson’s disease (PD; Coundouris et al., 2020). Several studies have demonstrated that older adults with clinical disorders such as very mild dementia and amnestic MCI (aMCI) are worse at performing PM tasks than healthy older adults (McDaniel et al., 2011; Niedźwieńska & Kvavilashvili, 2014; Shelton et al., 2016). It has also been shown that the PM of older adults with Alzheimer’s disease (AD) is worse than that of older adults with aMCI (Troyer & Murphy, 2007), indicating that ageing and a decline in cognitive status result in a more profound PM deficit. A recent review reported that the PM impairment observed in a clinical population could either result from the disruption of key neurocognitive resources (e.g., attentional capacity, processing speed, executive control, metacognition, and working memory) or be independent of these resources (Henry, 2021). Limited attention and executive dysfunction are prevalent symptoms of age-related clinical disorders such as AD (Kobeleva et al., 2017), stroke (Hayes et al., 2011) and PD (Uc et al., 2007). Therefore, the clinical older population might experience PM difficulties due to limited cognitive resources as a result of normal ageing or due to failures in planning and executing PM intentions.

Both healthy and clinical older populations face PM difficulties. However, we currently lack a standardised method of alleviating PM impairments in older populations. Therefore, this review attempts to inform the field by systematically summarising the current PM training approaches used for healthy and clinical older populations.

A number of assessment tools have been developed to diagnose, assess, and evaluate the effects of different treatments on PM. Laboratory PM tasks (e.g., lexical decision task in Ihle et al., 2018; two-back working memory task in Zöllig et al., 2012) and naturalistic PM tasks (e.g., telephone call task in Troyer, 2001; writing the day of the week in Burkard et al., 2014b) are commonly used to measure PM performance in older adults. Validated objective PM assessments (e.g., the Cambridge Test of Prospective Memory [CAMPROMPT]; Wilson et al., 2005) and self-reported questionnaires (e.g., Prospective and Retrospective Memory Questionnaire [PRMQ]; Crawford et al., 2003; Comprehensive Assessment of Prospective Memory [CAPM]; Chau et al., 2007) are also used to diagnose and evaluate PM impairment, respectively.

Although older adults often report PM impairment, some studies have documented a contradictory phenomenon called the age–PM paradox (Rendell & Craik, 2000; Schnitzspahn et al., 2011), wherein younger adults outperform older adults only in the laboratory PM task but not in the naturalistic task (Henry et al., 2004). However, recent studies that have revisited the age–PM paradox suggest that the benefits conferred by older age on naturalistic PM tasks may have been overestimated (Hering, Cortez, et al., 2014; Koo et al., 2021; Schnitzspahn et al., 2020). In fact, the older adults likely perform neither significantly better nor worse than younger adults in the naturalistic PM tasks (Schnitzspahn et al., 2020).

PM is important for everyday functioning in older adults. PM, temporal order memory and source memory play important roles in supporting instrumental activities of daily living (IADLs; Schmitter-Edgecombe et al., 2009). Recently, a PM computerised measure, the Virtual Week, was found to be positively correlated with the IADLs (Hering et al., 2018). Both TBPM and EBPM have been found to be significant mediators of the relationship between age and everyday functioning (Sheppard et al., 2020). Previous research indicates that poor PM performance can predict a poor performance in the day-out task, which is a naturalistic task used to examine everyday functioning in individuals with MCI (Schmitter-Edgecombe et al., 2012). Inferior PM performance and self-reported PM failures are also associated with a lower quality of life in older adults (Woods et al., 2015). Given the consequences of PM decline and its relevance to independent functioning, it is essential to explore and review training regimes or interventions that can enhance PM performance in older adults.

Cognitive interventions can help enhance the cognitive functions of older adults. A two-year multi-domain intervention (diet, exercise, cognitive training, and vascular risk monitoring) has been found to either improve or maintain cognitive functioning in at-risk older adults in domains such as executive function, processing speed, and memory (Ngandu et al., 2015). Therefore, it has been suggested that PM training, when provided as a cognitive intervention, can help slow down PM decline in older adults. Kliegel et al. (2011) proposed a theory-driven training approach for improving PM in people with PD, with the PM training categorised into four phases or processes: (1) intention formation, (2) intention retention, (3) intention initiation, and (4) intention execution. They postulated different mechanisms underlying different PM phases, such as the involvement of planning in the intention formation stage and the involvement of retrospective memory in the intention retention phase. Given that some of these phases are more likely to be affected in people with PD, effective training approaches are required to target specific PM phases.

Aside from the theory-driven training approach, Hering et al. (2014) also outlined two possible PM training approaches for older adults: (1) strategy-oriented training and (2) process-based training. The former aims to compensate for the loss of PM and the latter aims to restore the cognitive processes surrounding PM.

Strategy-based training involves the use of internal or external mnemonic strategies to compensate for any PM-based difficulties. For example, mnemonic strategies such as imagery and rehearsal training can help improve PM intention formation and retention, respectively (Ihle et al., 2018). A meta-analysis by Chen et al. (2015) reported that implementation intention, an ‘if–then’ technique used to strengthen the cue–response association, can effectively improve PM in both younger (*d* = 0.45) and older adults (*d* = 0.68). This meta-analysis supports strategy-based PM training regimes that use a simple internal mnemonic technique and ask participants to encode PM in the form of ‘if situation Y is encountered, then I will initiate the goal-directed behaviour X’. Another strategy-based PM training regime that incorporates both internal and external mnemonic strategy training (e.g., use of an agenda and elaborated imagery) has also demonstrated gains in enhancing PM in older adults (Schmidt et al., 2001). In addition to healthy older adults, strategy-based training also facilitates PM improvement in older adults with clinical disorders. A memory rehabilitation intervention that highlights strategies for everyday tasks has been shown to effectively improve PM in older adults with either MCI or impaired memory (Kinsella et al., 2009; Mateos et al., 2016). A spaced retrieval training that emphasises successful recall with extended intermittent times was also shown to improve PM in older adults with MCI (Ozgis et al., 2009). Implementation intention also effectively improved PM in older adults with mild AD (Lee et al., 2016). In fact, strategy use has been positively associated with better PM in both healthy older adults and those with clinical disorders (Hutchens et al., 2012; Kinsella et al., 2009). Teaching and practicing the use of mnemonic strategies can also boost PM performance. All of these findings suggest that PM can be improved with a higher frequency of strategy use.

In contrast, process-based training comprises repeated and intensive exercise of PM. The level of difficulty is increased stepwise during the training. For instance, process-based training regimes such as Virtual Week and errorless learning-based memory training improved PM in healthy older adults and in those with early AD, respectively (Lee et al., 2013; Rose et al., 2015). The healthy older adults were trained on a Virtual Week over 12 one-hour sessions for a month (Rose et al., 2015). In the Virtual Week, participants were simultaneously asked to perform regular and irregular EBPM and TBPM tasks whose difficulty increased over time. For example, a regular TBPM task asked participants to select the correct task from a list at a specific time during the game. The level of difficulty was adjusted based on the number and complexity of the PM tasks. The behavioural results illustrated that training gains were achieved not only in the trained task but also in untrained tasks such as call-back task and timed instrumental activities of daily living (TIADL). Lee et al. (2013) offered an errorless learning-based memory training regime to older adults with early AD over 12 30-min sessions for 6 weeks. Sensory memory, working memory, PM and memory strategies were trained in a non-threatening training atmosphere using small components, practice, feedback and spaced retrieval strategies. After training, participants in the computer-assisted group demonstrated better PM performance and everyday functioning. The findings of these studies suggest that process-based training may be effective in improving PM in healthy older adults and in those with clinical disorders. However, as mentioned in Hering, Rendell, et al. (2014), there is limited literature on PM process-based training. More studies are required in this field to better understand the benefits of such training regimes in improving PM and everyday functioning.

Hering et al. (2014) provided a comprehensive summary of current PM training regimes that use strategy-based and process-based training approaches, in which two approaches are commonly used in the literature to classify PM training. Nevertheless, it should be pointed out that this classification is arbitrary and not absolute. Another meta-analysis by Chen et al. (2015) also reiterated the training gains of implementation intentions on PM in older adults. Although several studies have investigated the effects of PM training in older adults, the methods and training approaches have varied across the studies. Hering et al. (2014) did not explicitly investigate the training gains in healthy and clinical populations, and Chen et al. (2015) only studied one of the abovementioned PM training regimes (i.e., implementation intentions). Therefore, there is a need to conduct a systematic review and meta-analysis to clearly summarise the evidence.

This review aims to address the above research gaps. First, previous studies have revealed mixed findings regarding long-term training efficacies (Emsaki et al., 2017; Insel et al., 2016; Kinsella et al., 2009; Lee et al., 2013). For example, PM training regimes such as memory intervention (Kinsella et al., 2009) have been shown to improve PM when assessed at post-test and 4-month follow-up. In contrast, errorless learning (Lee et al., 2013) and multifaceted prospective memory interventions (Insel et al., 2016) have reported either negligible gains or even loss of training gains after 3 or 5 months, respectively. Therefore, to clarify these discrepancies and provide reliable results, it is valuable to examine the immediate and long-term efficacies of these regimes. Second, previous reviews have not separately investigated the training effects in healthy and clinical populations (Chen et al., 2015; Hering et al., 2014). Prior research has shown that implementation intentions can improve PM in healthy younger adults, healthy older adults and participants with multiple sclerosis, brain injuries, schizotypal features and autism spectrum disorders (Chen et al., 2015). However, the existing literature does not address whether training can alleviate PM impairment in older clinical groups, such as those with MCI, dementia, stroke and PD. Therefore, this review independently probes the training effects on these populations. Third, although both healthy and clinical older adults experience PM impairment, the former might have a milder impairment (i.e., age-related PM decline), whereas the latter might have a more significant impairment (i.e., PM deficit). To expand the current literature beyond the well-known strategy-based training (e.g., implementation intentions), this review examines whether process-based training for older adults (e.g., spaced retrieval or errorless learning, as proposed in Hering, Rendell, et al., 2014) is helpful in alleviating PM decline and deficit. While most strategy-based regimes consist of single-session training, most process-based regimes include programme-based training. It is therefore useful to study the variations that arise from different training durations. This review explores PM training efficacy over time (immediate or long-term efficacy), in different study populations (healthy or clinical population), for different types of training (strategy- or process-based approach) and over different training durations.

We hypothesise that PM training will have an overall efficacy. In addition, four exploratory hypotheses are proposed. Regarding the mixed evidence on the immediate and long-term effects of training, we anticipate that immediate efficacy will be greater than long-term efficacy as the long-term training effect can fade over time. If the overall efficacy of training is found to be heterogeneous, three subgroup analyses will be conducted. As the clinical population is likely to have more room for improvement, we predict a larger effect for the clinical population than for the healthy population. As process-based training focuses on detailed procedures and a stepwise approach, we expect a larger effect for process-based training than for strategy-based training. With respect to the training duration, we anticipate that longer durations will have stronger effects as the trainee will receive more input over longer durations. The proper testing of these hypotheses will depend on the number of studies conducted under various conditions.

## Methods

The conduct and reporting of this review was in accordance with the Preferred Reporting Items for Systematic Reviews and Meta-Analyses (PRISMA) statement (Moher et al., 2009) and Cochrane handbook for systematic reviews of interventions (Higgins & Green, 2011). The review has also been registered in the Prospective Register of Systematic Reviews (PROSPERO) (https://www.crd.york.ac.uk/prospero/display_record.php?ID=CRD42020167986).

### Search Strategy

Systematic electronic searches were conducted in the following databases from inception till 29 October 2020: Cochrane Library, PsycInfo, PubMed, CINAHL, EMBASE, Web of Sciences and Scopus. The key search terms were synonyms of (1) ‘prospective memory’ AND (2) ‘older adults’ AND (3) ‘intervention’ OR ‘training’. Subject heading searches were also conducted where possible. The searches were limited to English peer-reviewed articles and those dealing with an older population, but not limited by publication dates. We excluded grey literature, case studies and other qualitative studies to provide better evidence of PM training. We only included quantitative and peer-reviewed articles as they offered most objective evidence of PM training. Though other qualitative studies made valuable contributions to the evidence on the efficacy of PM training, we focused on the quantitative findings and statistical evidence of PM training. All of the references and citations listed in the eligible articles were also searched. A meta-analysis paper on implementation intention (Chen et al., 2015) was also included and supplemented by an updated search. An example of the search strategy is shown in Appendix A.

### Selection Criteria

#### Types of Participants

As done for other similar systematic reviews and meta-analyses (Floyd & Scogin, 1997; Gross et al., 2012; Martin et al., 2011; Nguyen et al., 2019), participants with a mean sample age ≥ 60 years were included. As this review aimed to study the efficacy of PM training across different populations, both healthy and clinical populations were included. The clinical population included older adults with a diagnosis of MCI, dementia, AD, stroke, PD, and TBI.

#### Types of Interventions

This review aimed to identify the current training options available to improve PM. Therefore, training programmes, interventions, rehabilitation programmes and encoding strategies for PM were included. However, studies that introduced general executive function training (e.g., shifting) and those that proposed interventions without empirical data were excluded.

#### Types of Comparators

Studies that applied (1) cross-sectional (i.e., comparison groups); (2) longitudinal (i.e., participants assessed before and after intervention) or (3) both cross-sectional and longitudinal designs were included. The comparison condition included but was not limited to active or passive control groups. Active controls consisted of alternative activities such as standard rehearsal encoding procedures (Ozgis et al., 2009), educational training (Schmidt et al., 2001) and life story interviews (Tappen & Hain, 2013). Passive controls included no contact or waitlist controls (Lee et al., 2013).

#### Types of Outcomes

Studies were included if they measured training outcomes with objective or self-reported PM measures for at least one-time point after training. Self-reported PM measures included questionnaires such as PRMQ and Brief Assessment of Prospective Memory (BAPM), as used in Zeintl et al. (2006) and Lee et al. (2013). The objective PM measures included PM laboratory tasks and neuropsychological assessments (e.g., CAMPROMPT; Gryffydd et al., 2020; Zeintl et al., 2006).

Studies were excluded if they included data reported in a previous study, or were case studies, qualitative studies, non-experimental designs, protocols, reviews, meta-analyses, dissertations, conference abstracts or proceedings, or were not peer-reviewed.

The initial search was conducted by the first author (Z.T.). Title and abstract screening and full-text screening were then independently conducted by two reviewers (Z.T. and D.N.). Consensus on article inclusion was reached by discussion if necessary.

### Methodological Quality

The methodological qualities of the eligible RCTs and non-RCTs were assessed using the revised Cochrane risk-of-bias tool for randomised trials (RoB 2; Sterne et al., 2019) and risk-of-bias in non-randomised studies – of interventions (ROBINS-I; Sterne et al., 2016), respectively. The methodological quality ratings were conducted by two independent reviewers (Z.T. and either D.N. or Y.C.) A consensus on the final ratings was reached by discussion.

### Data Extraction

#### Systematic Review

The study characteristics and findings from all eligible studies, including RCTs and non-RCTs, were extracted using a comprehensive data collection form in Excel. The information was summarised in a table and qualitatively synthesised in text.

#### Meta-analysis

Only RCTs were included in the meta-analysis as they had better quality of evidence (Ahn & Kang, 2018). Two independent reviewers (Z.T. and either D.N. or Y.C.) agreed on the categorisation of RCTs and non-RCTs.

The data extraction for meta-analysis was coded in Excel by two independent reviewers (Z.T. and either D.N. or Y.C.). Data on older adults (i.e., means, standard deviations [*SD*s], sample sizes of training and control groups, *M*_change_, proportion of corrected response, *t*-value, *F*-value, *p*-value and Cohen’s *d*), as examined by PM measures, were extracted. Raw data were converted, if necessary, by formulae listed in the Cochrane handbook (e.g., *SE*s to *SD*s; Higgins & Green, 2011).

An attempt was made to contact authors to obtain additional information for effect size calculation. Nineteen authors were contacted, of whom four replied with sufficient data. These papers were included in the meta-analysis.

### Data Handling

The included studies employed multiple samples, measurement time-points, interventions, controls, or outcomes. Effect sizes were calculated based on the different conditions.

#### Studies with Multiple Samples

Data were extracted separately for healthy and clinical samples. The healthy older adults were community-dwelling and either from memory clinics without any clinical diagnosis or with memory complaints. The clinical populations were older adults with clinical diagnosis such as AD, mild AD and PD. The effect sizes were calculated separately.^1^

#### Studies with Multiple Time-Points

Data were extracted from the pre-test, post-test and follow-up periods. If both the baseline and pre-test data were available, only the pre-test data were extracted. The effect sizes were computed separately for comparisons between (1) the pre-test and post-test periods and (2) the pre-test and follow-up periods.

#### Studies with Multiple Interventions/Comparators

For studies that had more than one intervention, data were extracted separately and the effect sizes were computed independently for each intervention.^2^ However, the effect sizes were pooled for studies that had multiple control groups.

#### Studies with Multiple Outcomes

Data were extracted as separate entries for each outcome measure from the same study. Multiple effect sizes from the same study were then pooled using the Comprehensive Meta-Analysis (CMA; v3.3; Borenstein et al., 2014) software. Therefore, only one effect size was derived from one study, except for studies assessing multiple time points and populations.

### Planned Data Analysis

CMA was used to perform the meta-analysis. Extracted data from Excel were used as inputs for CMA. Hedges’ *g* was calculated for the effect size to account for the small sample size bias. A positive Hedges’ *g* indicated a positive training effect. A Hedges’ *g* of 0.2, 0.5 or 0.8 was interpreted as a small, moderate and large effect, respectively (Cohen, 2013). The calculated Hedges’ *g* was cross-checked by another author (J.M.O.).

None of the studies showed correlations between the pre-test and post-test measures. Borenstein et al. (2011) suggested that correlations could be estimated from related studies. Therefore, following the procedure used by another meta-analysis of executive function training (Nguyen et al., 2019), the current study imputed 0.60 as the pre-post correlation for all of the studies.

The effectiveness of the PM training was investigated by examining the immediate and long-term efficacies separately. The immediate efficacy investigated the performance difference between the pre-test and post-test, whereas the long-term efficacy assessed the performance difference between the pre-test and follow-up.

#### Moderator Analyses

In case of high heterogeneity, planned subgroup meta-analyses were performed to investigate the effects of moderators, namely, (1) study population, (2) types of training, and (3) training duration.

#### Heterogeneity and Publication Bias

Heterogeneity was assessed by *Q* value, *I*^2^ value, tau and tau square. A significant *Q* statistic and larger *I*^2^ value indicated that the true variance could be explained by other moderators (Borenstein et al., 2011). An *I*^2^ value of 25%, 50% and 75% was interpreted as minor, moderate and high heterogeneity, respectively (Higgins et al., 2003). In accordance with the Cochrane handbook (Higgins & Green, 2011) and two Cochrane reviews on cognitive or memory rehabilitation (das Nair et al., 2016a, 2016b), the use of fixed-effects or random-effects models depended on the heterogeneity of the data.

Publication bias was investigated using (1) funnel plots by Hedges’ *g*, (2) Egger’s regression intercept, and (3) Duval and Tweedie’s trim-and-fill method.

#### Sensitivity Analysis

To check the consistency of results across different methods, sensitivity analyses were conducted on (1) the risk of bias (i.e., low level, some concerns, and high level), (2) study design (i.e., cross-sectional, longitudinal, and both cross-sectional and longitudinal), and (3) different pre–post correlation values (*r* = 0.00, *r* = 0.60 and *r* = 0.90).

## Results

### Search Results

**Fig. 1:**
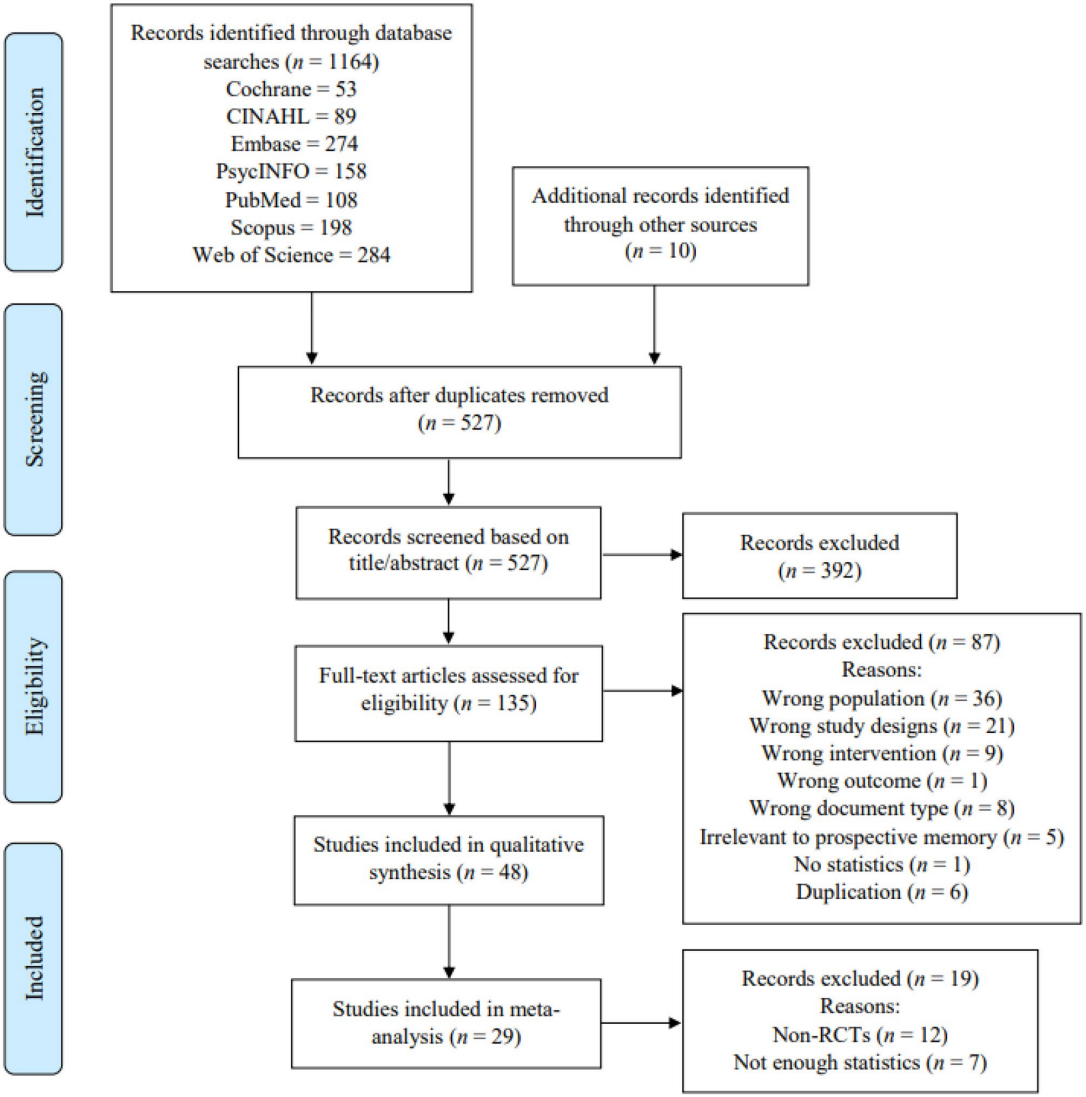
PRISMA flow chart of the study selection process

The current review comprises two parts, including qualitative synthesis and meta-analysis. The search process is detailed in the flowchart shown in Fig. 1. Our search yielded 1,174 articles, which reduced to 527 after removing duplicates. After the title and abstract screening, 135 articles remained for full-text screening. A further 87 were excluded by two reviewers, resulting in a total of 48 articles that were eligible for qualitative synthesis. After excluding the non-RCTs and RCTs with insufficient data, 29 studies were included in the meta-analysis.

We found excellent inter-rater agreement between the reviewers for article inclusion (94%). A consensus was reached by discussing the basis for inclusion or exclusion of each study. The most common reasons for exclusion were wrong population group, improper study design and irrelevant training.

### Qualitative Synthesis

#### Characteristics of Included Studies

Fifteen studies were conducted in the USA (31%), seven in Australia (15%), six in Switzerland (13%), three each in Germany (6%) and Canada (6%), two each in Greece (4%) and the UK (4%), and one each in Hong Kong (2%), Iran (2%), Malaysia (2%), Poland (2%), Portugal (2%), Spain (2%), the Netherlands (2%) and Italy (2%).

Appendices B and C summarise the study characteristics, designs, training, outcomes and findings (*n* = 48). Appendix D documents the overall effect size of each study.

#### Participants

In total, there were 2,957 older adults across 48 studies. However, six of these studies did not report sufficient demographic statistics (Andrewes et al., 1996; Burkard et al., 2014a; Cavallini et al., 2003; Chasteen et al., 2001; Ozgis et al., 2009; Rose et al., 2015). After excluding these studies, we had 2,672 participants across 42 studies with enough details to calculate the average demographic statistics (weighted *M*_age_ = 72.01 years, *SD* = 6.45, range = 50–94).^3^ An additional 12 studies did not report sufficient data for calculating the number of participants in the training and control conditions separately^4^ (Altgassen et al., 2015; Kinsella et al., 2016; Lee et al., 2013; McDougall, 2000; McFarland & Glisky, 2011; Niedźwieńska et al., 2014; Pereira et al., 2012, 2015, 2018; Schnitzspahn & Kliegel, 2009; Villa & Abeles, 2000; Zimmermann & Meier, 2010). As a result, the remaining 30 included studies^5^ had 993 participants (weighted *M*_age_ = 72.13 years, *SD* = 6.44, range = 50–93) in the training condition and 698 participants (weighted *M*_age_ = 71.48 years, *SD* = 6.42, range = 50–94) in the control condition.

#### PM Measures

Most of the PM measures could be classified as (1) naturalistic PM task, (2) laboratory PM task, (3) objective PM measure, and (4) self-reported PM measure. For example, one of the naturalistic PM tasks included remembering to return a form after the experiment and remembering to write their first names on the envelope. The laboratory PM tasks included computerised PM tasks in the dual-task paradigm, such as the lexical-decision and *n*-back tasks. The objective PM measures included objective neuropsychological measures such as CAMPROMPT and the Rivermead Behavioral Memory Test. Lastly, the self-reported measures included PRMQ, BAPM and the Multifactorial Memory Questionnaire (MMQ) – ability score.

#### Study Designs and Training

The included studies comprised 36 RCTs and 12 non-RCTs. Most of the studies (23/48) used a mixed design (i.e., both cross-sectional and longitudinal), 21/48 studies applied a cross-sectional design, and 4/48 studies applied a longitudinal design. Eleven studies involved at least one follow-up assessment after the PM training (Andrewes et al., 1996; Burkard et al., 2014a; Emsaki et al., 2017; Farzin et al., 2018; Insel et al., 2016; Kinsella et al., 2009, 2016; Lee et al., 2013; Schmidt et al., 1999, 2001; Troyer et al., 2008). The follow-up length ranged from four weeks to six months (*M* = 3.27 months, *SD* = 1.39).^6^,^7^

#### Training/Interventions

Fifty-eight interventions were extracted from the 48 included studies. Seven studies used two interventions (Chasteen et al., 2001; Lee et al., 2013; Niedźwieńska et al., 2014; Rebok et al., 1997; Schnitzspahn & Kliegel, 2009; Tsantali et al., 2017; Villa & Abeles, 2000) and one study used four interventions (Henry et al., 2020). Cavallini et al. (2003) also used two interventions (i.e., loci mnemonic and strategic training), but separate data for each intervention were unavailable. Analysis of variance (ANOVA) did not reveal any significant differences in the training effect between the loci mnemonic and strategic training interventions (*p* > 0.05).

Most of the PM training regimes belonged to the strategy-based training category (*n* = 49). The remaining interventions were classified as either process-based training (*n* = 3; Lee et al., 2013; Rose et al., 2015) or mixed strategy-based and process-based training (*n* = 6; Emsaki et al., 2017; Farzin et al., 2018; Insel et al., 2016; McDaniel et al., 2014; Poptsi et al., 2017; Waldum et al., 2016).

These 58 interventions were further broken down into single-session (*n* = 29) and programme-based training (*n* = 29). The single-session PM training regimes all used strategy-based training, which included implementation intention (*n* = 20), enactment encoding (*n* = 3), future thinking and planning (*n* = 2), feedback provision during a PM task (*n* = 2), sequence learning intervention (*n* = 1), and spaced retrieval (*n* = 1).

There were a lot of variations in the programme-based PM training interventions, which included strategy-based (*n* = 20), process-based (*n* = 3), and mixed strategy- and process-based training (*n* = 6). They also included cognitive training and mnemonic psychoeducation. The intervention length ranged from 3 weeks to 2 years (*M* = 10.96 months, *SD* = 19.62).^8^ The average number of sessions was 14.57 (*SD* = 17.17, range = 4–80).^9^ The frequency of training ranged from one session per week to three sessions per week (*M* = 1.74, *SD* = 0.75),^10^ with the session durations ranging from 30 to 120 min (*M* = 79.54 min, *SD* = 31.20).^11^

Table 1 shows a summary of the interventions.

#### Overall PM Training Efficacy

Overall, 43% of the 58 interventions (*n* = 25) reported positive findings, whereas 36% reported mixed results (*n* = 21). The remaining interventions demonstrated no significant improvement (*n* = 11; 19%) and negative results (*n* = 1; 2%).

Of the 25 effective PM training interventions, 21 used strategy-based training, one used process-based training (Lee et al., 2013) and three used mixed approaches (Farzin et al., 2018; Insel et al., 2016; McDaniel et al., 2014). Most of the effective PM training regimes consisted of cognitive and strategy-based memory training (*n* = 11), and the remaining mainly focused on encoding strategies such as implementation intention (*n* = 8), enactment (*n* = 3), planning or future thinking (*n* = 2), and spaced retrieval (*n* = 1).

Of the 21 interventions that reported mixed results, 17 used strategy-based training, one used process-based training (Rose et al., 2015) and three used mixed approaches (Farzin et al., 2018; Poptsi et al., 2017; Waldum et al., 2016). Most of the interventions were categorised as memory or cognitive training (*n* = 10), including three PM-specific training regimes. Some encoding strategies, such as implementation intention (*n* = 7), feedback provision (*n* = 2) and imagery training (*n* = 2), also reported mixed results.

Eleven interventions showed no significant improvement after training, of which 10 used strategy-based training and one used process-based training (Lee et al., 2013). Nearly half of these interventions consisted of memory or cognitive training regimes (*n* = 6), and the other half used implementation intention (*n* = 5).

Only one study revealed a negative training efficacy after delivering a memory specificity training, which was a mixed-approach intervention (Emsaki et al., 2017). Further quantitative analyses were performed in the meta-analysis.

#### Methodological Quality

There was substantial inter-rater reliability regarding the ratings of methodological quality (average κ = 0.62, *p* < 0.001). A consensus on the final ratings was reached via discussion.

Based on the RoB 2.0, 12 RCTs (33%) had a low risk of bias. Sixteen RCTs (45%) were rated as having some concern, and eight RCTs (22%) were rated has having a high risk of bias. Across the five domains, only 75% of the RCTs were rated as having a low risk of bias arising from randomisation, whereas 64% of the RCTs had a low risk of bias due to deviations from the intended interventions. Most of the RCTs (92%) were rated as having a low risk of bias due to missing outcome data, 75% and 83% of the RCTs had a low risk of bias in terms of measurement of outcomes and selection of the reported results, respectively.

The ROBINS-I revealed three non-RCTs (25%) with a low risk of bias, four (34%) with a moderate risk of bias, four (33%) with a serious risk of bias, and one (8%) with a critical risk of bias. Only 33% of the non-RCTs were rated as having a low risk of bias due to confounding. The study quality was sound in other domains, with the majority of the non-RCTs having a low risk of bias in terms of selection of participants (83%), classification of interventions (100%), deviations from the intended interventions (83%), missing data (67%), measurement of outcomes (58%), and selection of the reported results (92%).

**Fig. 2:**
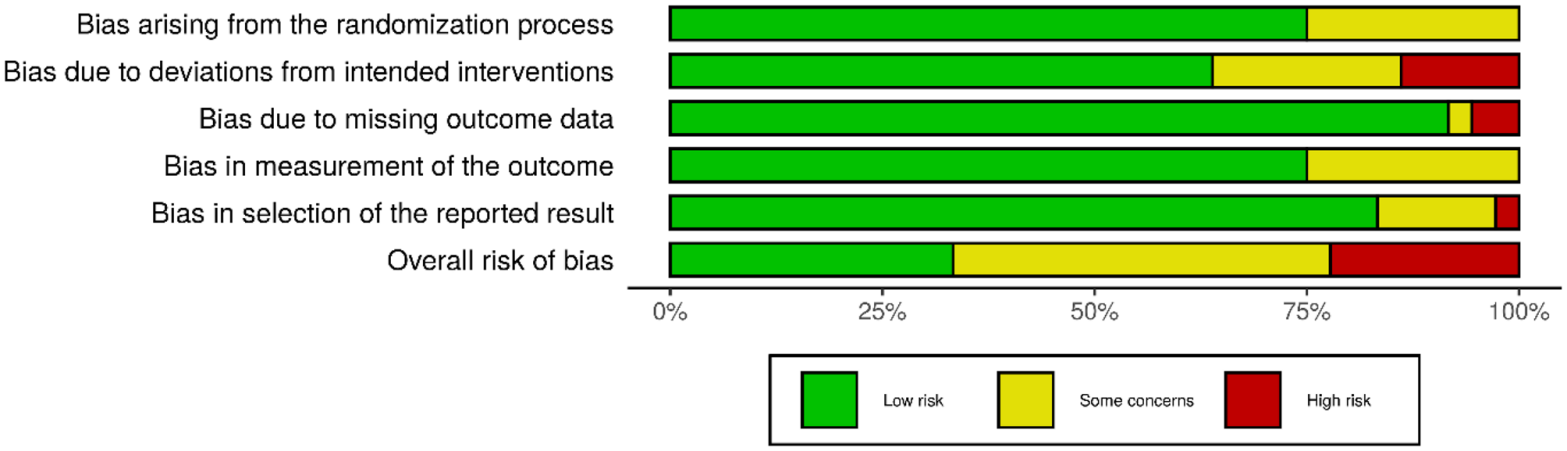
Weighted summary of RCTs. Figure generated by Risk‐of‐bias VISualization (robvis; McGuinness & Higgins, 2020)

**Fig. 3:**
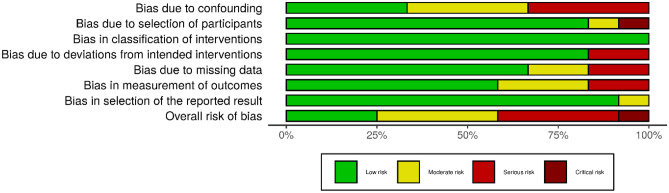
Weighted summary of non-RCTs. Figure generated by Risk‐of‐bias VISualization (robvis; McGuinness & Higgins, 2020)

**Fig. 4:**
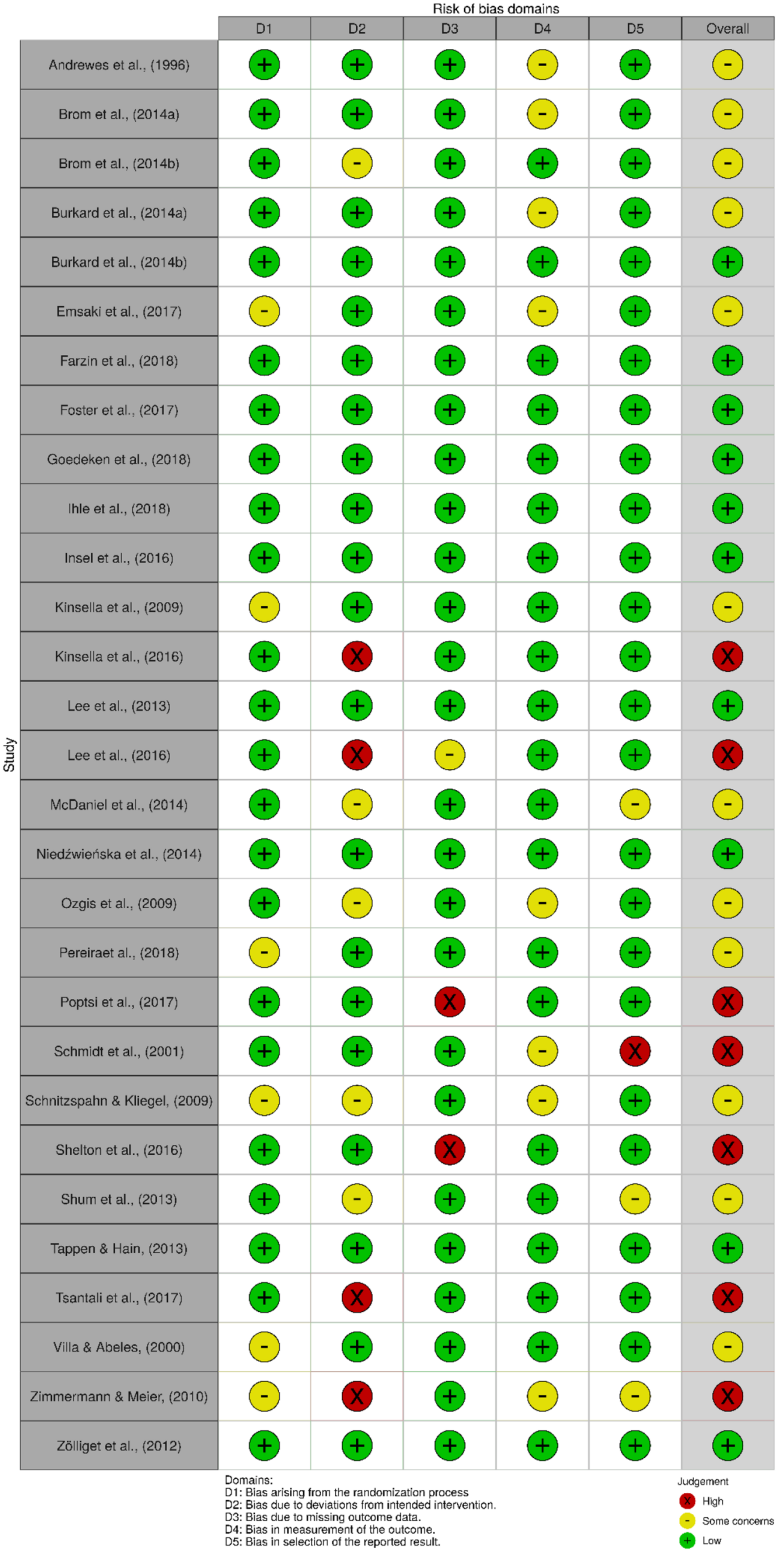
Summary of the methodology quality of RCTs rated in each study. Figure generated by Risk‐of‐bias VISualization (robvis; McGuinness & Higgins, 2020)

**Fig. 5:**
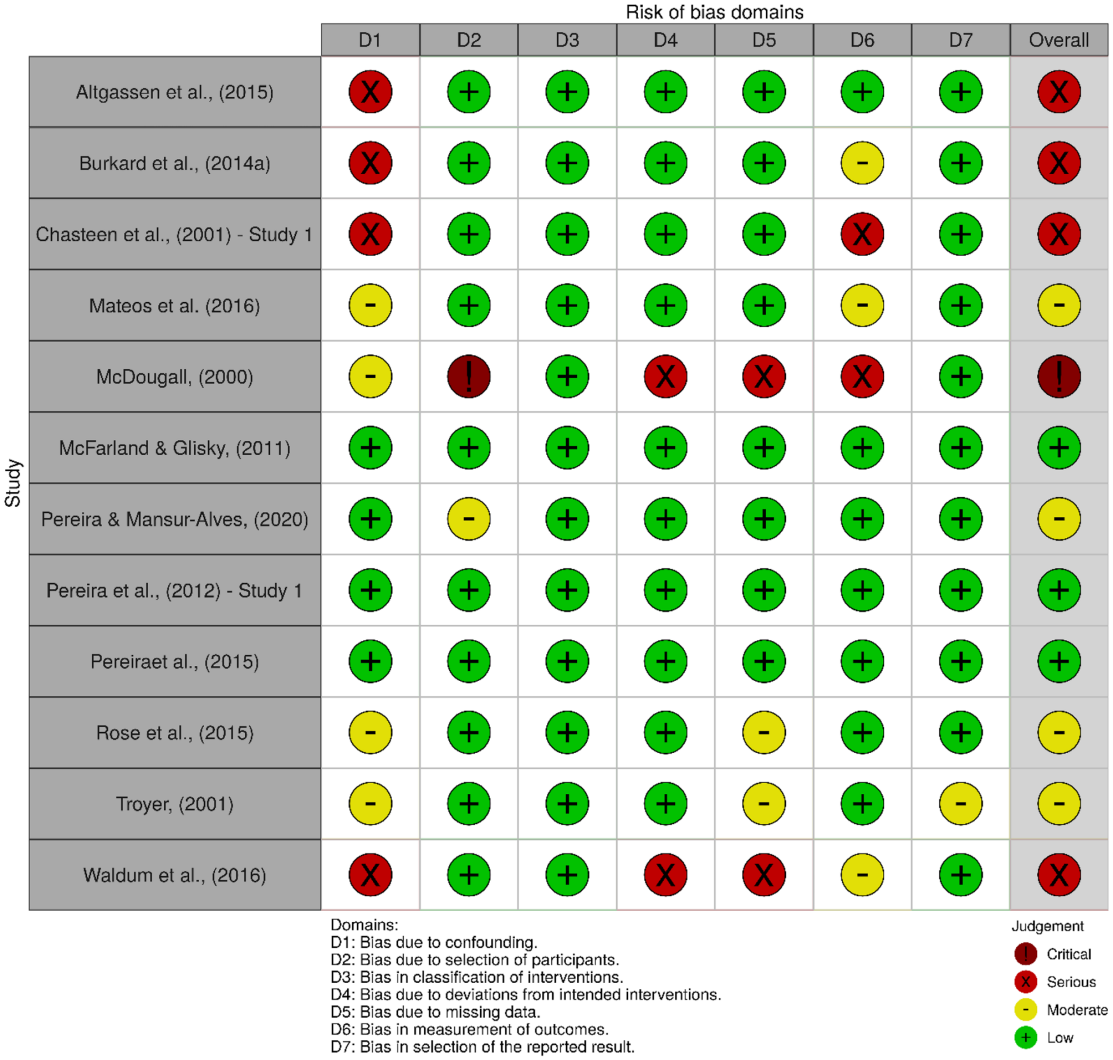
Summary of the methodology quality of non-RCTs rated in each study. Figure generated by Risk‐of‐bias VISualization (robvis; McGuinness & Higgins, 2020)

Figures 2 and 3 show the weighted summary of the methodological quality rated by RoB 2.0 and ROBINS-I, respectively. Figures 4 and 5 show the summary of the methodological quality in each study rated by RoB 2.0 and ROBINS-I, respectively.

### Meta-analysis

#### Participants and Study Designs

Twenty-nine studies were eligible for inclusion in the subsequent meta-analysis. Of the 48 studies originally included, 19 were excluded as they were either non-RCTs (*n* = 12) or did not report sufficient or relevant data for calculation of the effect size (*n* = 7).

#### Immediate Efficacy

**Fig. 6:**
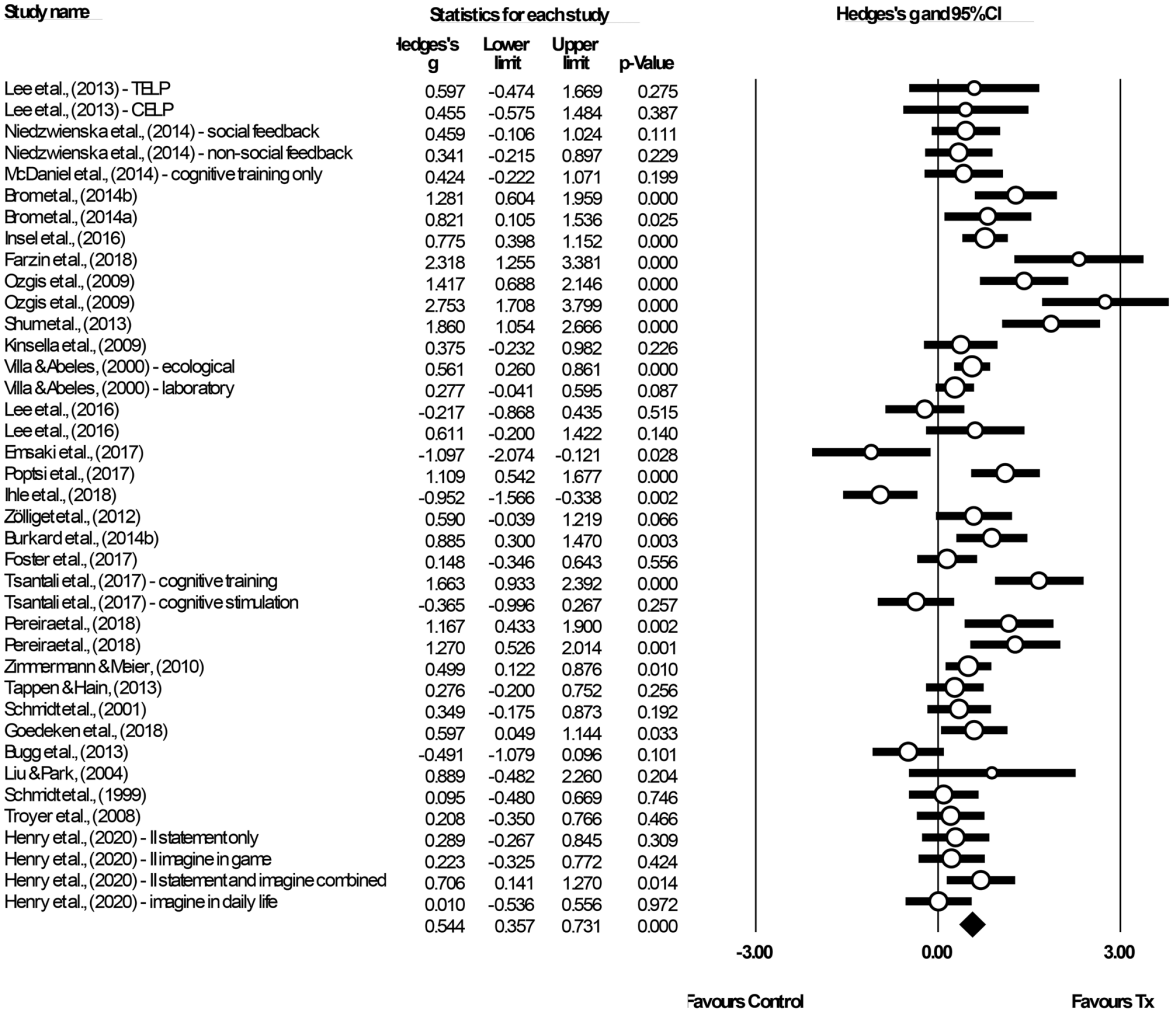
Forest plot showing individual and summary effect size for immediate efficacy

**Fig. 7:**
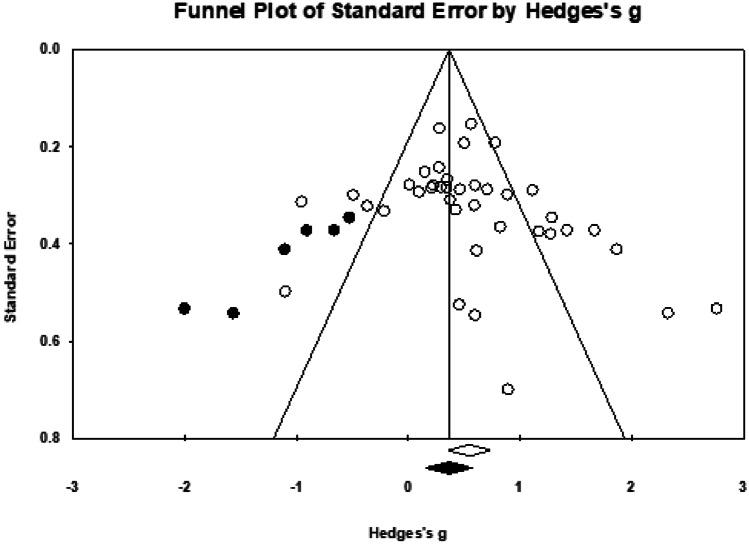
Funnel plot examining the publication bias. Imputed point estimated from the trim-and-fill analysis were shaded in black

The studies included in the meta-analysis of immediate efficacy had a total of 1,629 older adults, of whom 881 were trained (*M*_sample size_ = 22.59, *SD* = 10.60) and 748 were control participants (*M*_sample size_ = 23.38, *SD* = 13.55).^12^ Thirty-six interventions were extracted from 29 studies (see Fig. 6), of which the majority used implementation intention (13/36). Given the variation across studies, a random-effects model was used. We calculated a significant, moderate mean effect size for the PM training, *g* = 0.54, 95% confidence interval (CI) [0.36, 0.73], *p* < 0.001, and a significant moderate-to-high heterogeneity among the studies, *Q* (38) = 145.99, *p* < 0.001, *I*^2^ = 73.97%, τ = 0.49 and τ^2^ = 0.24. The prediction interval was -0.47–1.56. The funnel plot showed an asymmetry that favoured a positive training efficacy (see Fig. 7). However, Egger’s test results were not significant (β = 1.50, *SE* = 0.99, *p* = 0.069). The trim-and-fill analysis was performed to adjust the potential publication bias, following which six studies with negative effect sizes were imputed, *g*_adjusted_ = 0.36, 95% CI [0.16, 0.57]. The likely impact of publication bias was considered to be modest.

**Fig. 8:**
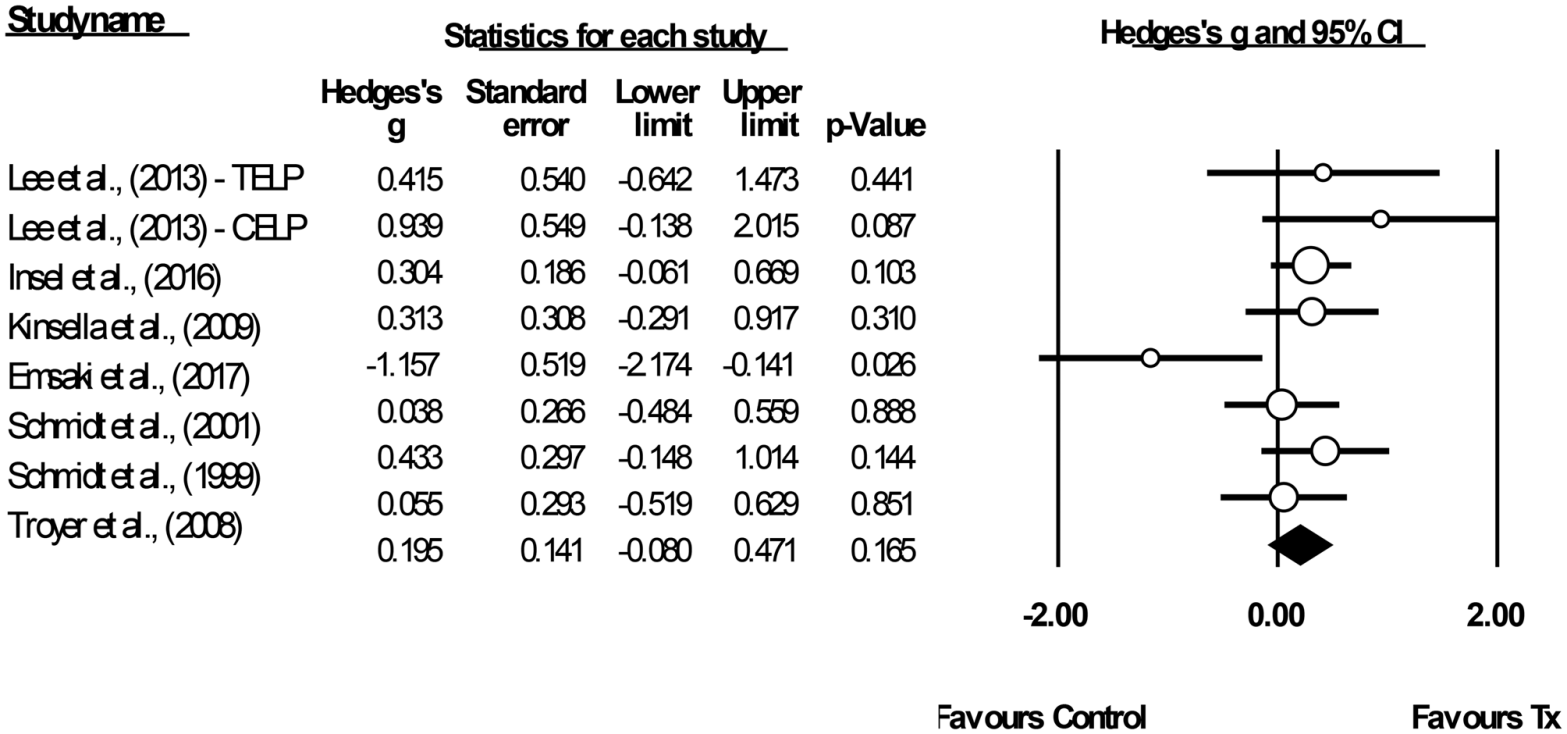
Forest plot showing individual and summary effect size for long-term efficacy

**Fig. 9:**
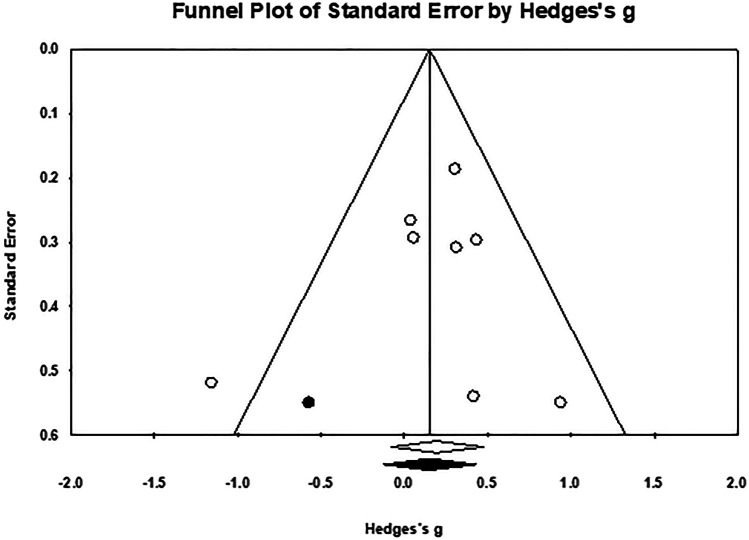
Funnel plot examining the publication bias. Imputed point estimated from the trim-and-fill analysis were shaded in black

#### Long-term Efficacy

The studies included in the meta-analysis of long-term efficacy had a total of 349 older adults, of whom 168 were trained (*M*_sample size_ = 21, *SD* = 16.66) and 181 were control participants (*M*_sample size_ = 25.86, *SD* = 18.85; see footnote 12). Eight interventions were extracted from seven studies (see Fig. 8), of which the majority conducted follow-up assessment at either three months (*n* = 5), four months (*n* = 1) or five months (*n* = 1). All of the interventions were programme-based, of which two were process-based, two were mixed strategy-based and process-based and four were strategy-based. Due to the variation across studies, a random-effects model was used. The mean effect size for the PM training was not significant, *g* = 0.20, 95% CI [-0.08, 0.47], *p* = 0.165, and neither was the heterogeneity, *Q* (7) = 10.49, *p* = 0.163, *I*^2^ = 33.26%, τ = 0.22 and τ^2^ = 0.05. The prediction interval was -0.45–0.84. The funnel plot showed an asymmetry that favoured a positive training efficacy (see Fig. 9). However, Egger’s test results were not significant (β = -0.58, *SE* = 1.34, *p* = 0.340). The trim-and-fill analysis was performed to adjust the potential publication bias, following which one study with negative effect size was imputed, *g*_adjusted_ = 0.15, 95% CI [-0.13, 0.43]. The likely impact of publication bias was considered to be trivial. 

#### Moderator Analyses

Subgroup analyses were conducted to further investigate the heterogeneity in effect sizes between studies. In accordance with the Cochrane handbook, at least 10 studies are required to justify subgroup analyses (Higgins & Green, 2011). As the long-term efficacy analysis included only eight interventions, we conducted moderator analyses only for immediate efficacy. Given the variation across studies (Borenstein, 2019), random-effects models were used.

First, due to the diverse nature of the clinical populations in the included studies, moderator analyses were not performed for separate clinical subgroups. Instead, as most of the clinical subgroups shared similar mechanisms (i.e., disruption of key neurocognitive resources; Henry, 2021), the moderator analysis integrated the subgroups from 18 studies with clinical populations. The training efficacy was not moderated by the study population (i.e., healthy and clinical populations), *Q* (1) = 0.04, *p* = 0.838, and both the healthy (*k* = 25, *g* = 0.53, *SE* = 0.12, *p* < 0.001) and clinical populations (*k* = 14, *g* = 0.57, *SE* = 0.17, *p* = 0.001) demonstrated significant positive training effects. The difference between the subgroups was 0.04, with 95% CI [-0.36, 0.44]. There was significant heterogeneity in the healthy population, *Q* (24) = 88.90, *p* < 0.001, *I*^2^ = 73.00%, τ = 0.45 and τ^2^ = 0.21. The prediction interval was -0.44–1.50. The heterogeneity was also significant for clinical population, *Q* (13) = 56.94, *p* < 0.001, *I*^2^ = 77.17%, τ = 0.62 and τ^2^ = 0.39. The prediction interval was -0.83–1.98.

Due to the limited number of studies that used process-based training (*n* = 3), we did not have the minimum number required to justify subgroup analyses. Therefore, in this review, we did not perform a subgroup analysis of the difference between strategy-based and process-based training. However, training duration can be used as a proxy for the training content, allowing us to conduct another subgroup analysis on training duration.

As the included studies reported a high variation in the training duration (i.e., number of sessions in training; *M*_sessions_ = 14.57, *SD* = 17.17, range = 4–80), the dichotomous variable of the number of sessions in training (i.e., programme-based or single-session) was evaluated. The subgroup analysis showed that this number was not a significant moderator, *Q* (1) = 1.95, *p* = 0.163. Further analysis revealed that both the programme-based (*k* = 17, *g* = 0.39, *SE* = 0.15, *p* = 0.007) and single-session trainings (*k* = 22, *g* = 0.66, *SE* = 0.13, *p* < 0.001) had a significantly positive training effect. The difference between subgroups was 0.27, with 95% CI [-0.11, 0.65]. There was significant heterogeneity among the studies with respect to programme-based training, *Q* (16) = 70.77, *p* < 0.001, *I*^2^ = 77.39%, τ = 0.51 and τ^2^ = 0.26. The prediction interval was -0.73–1.52. The heterogeneity was also significant among the studies for single-session training, *Q* (21) = 72.40, *p* < 0.001, *I*^2^ = 70.99%, τ = 0.49 and τ^2^ = 0.24. The prediction interval was -0.39–1.72. The summary and overview of the moderator effect sizes are shown in the Table 2.

#### Sensitivity Analysis

The risk of bias of the included studies showed negligible impact on the training efficacy, which was confirmed by the significant Hedges’ *g*. However, different study designs have illustrated some of the impacts on training efficacy. Although our analysis found a significant Hedges’ *g* for the cross-sectional and cross-sectional and longitudinal study designs, the Hedges’ *g* of the longitudinal design was not significant. Nevertheless, the limited number of included interventions with the longitudinal design (*n* = 2) precluded in-depth analysis.

The different values of the pre–post correlations also indicated minimal impact on training efficacy, with the immediate efficacy retaining a medium, significant effect size and the long-term efficacy retaining a small effect size. Although the effect sizes of long-term efficacy became significant when *r* = 0.90, the number of included interventions were small (*n* = 8). Thus, the data need to be interpreted with caution. Appendix E includes the summary results of the sensitivity analyses.

## Discussion

This systematic review and meta-analysis investigated the efficacy of PM training in older adults. Forty-eight studies (36 RCTs and 12 non-RCTs) were included in the systematic review. However, as the methodological quality of the included studies varied across the RCTs and non-RCTs, only 29 RCTs were included in the subsequent meta-analysis. In the 48 studies, both qualitative and quantitative results showed promising efficacy of PM training. In the systematic review, 43% of the interventions demonstrated positive training results. In the meta-analysis, PM training was found to have a significant moderate immediate efficacy (*g* = 0.54) in improving PM in older adults, but no significant long-term efficacy (*g* = 0.20). This review also examined the potential moderators of training efficacy with respect to (1) study populations (i.e., healthy or clinical populations), (2) types of training (i.e., strategy-based or process-based training), and (3) the number of sessions in training (i.e., programme-based or single-session). Our analysis supported the exploratory hypothesis on training efficacy over time, but not the exploratory hypotheses on study population and training duration. Further subgroup analyses revealed significant training efficacy in both healthy and clinical populations, and significant training gains after both programme-based and single-session training. Due to the limited number of process-based training studies, the exploratory hypothesis on the types of training (i.e., strategy-based or process-based training) could not be tested. Overall, the findings filled an existing knowledge gap by systematically and comprehensively summarising PM training studies and providing some positive evidence to support the practice of PM training.

### PM Training Efficacy

#### Qualitative Synthesis

Overall, 25 of the 58 PM training regimes revealed significant positive training gains. PM training regimes that comprised cognitive training, strategic memory training and encoding strategies such as implementation intention and enactment improved PM in older adults. Consistent with the current literature, cognitive and strategic memory training regimes that involved metacognitive factors and internal and external mnemonic techniques were found to be effective in enhancing PM performance.

It has been suggested that PM training with mnemonic techniques can facilitate PM performance by teaching either methods of using external aids or strategies to develop better attentional abilities and improve the capacity for memory retention and retrieval (Mateos et al., 2016). Additionally, these strategies could compensate for the PM decline in older adults. Extensive practice of mnemonic strategies can help ameliorate such impairments in cognitive abilities. In line with current studies, training based on encoding strategies facilitates PM by strengthening the cue–response association. For instance, implementation intention can provide a more elaborative encoding of PM intentions by forming a mental representation of the task, thereby either increasing salience of the retrieval cues or enhancing the retrospective component of the task (Henry et al., 2020; Shelton et al., 2016). In keeping with the strategic attentional resources required for PM, encoding strategies such as enactment, planning, and spaced retrieval can increase the allocation of attentional resources to support and improve PM performance (Harrison & Einstein, 2010).

Only one study showed negative results of PM training (Emsaki et al., 2017). Although the study claimed a positive training effect, it recorded an elevated PRMQ score after training, indicating more PM failures. This contradiction between the descriptions and results might have occurred due to misinterpretation of the data.

It should be noted that 36% and 19% of the training interventions displayed either mixed or no significant findings, respectively. In fact, some studies have used more than one PM measure or measured more than one type of PM (i.e., TBPM and EBPM). As an example, Tappen and Hain (2013)’s study showed that in-home cognitive training only improved the performance of a simple EMPM task (i.e., remembering to take cash from an envelope), but not of a more complex EBPM task (i.e., remembering to get a bottle of water from refrigerator). Additionally, although providing social feedback effectively improved TBPM and irregular EBPM, it did not improve regular EBPM (Niedźwieńska et al., 2014). In contrast, Henry et al. (2020) found that implementation intention enhanced EBPM but not TBPM. In light of these multiple PM measures, the mixed findings do not come as a surprise. Another possible explanation for the mixed results might be the small sample sizes of the studies. From the statistical perspective, it would be hard to find a significant difference with a limited sample size. Therefore, a meta-analysis was performed to further evaluate the statistical power of each study.

#### Meta-analysis

The present meta-analysis found a significant moderate effect size for the immediate efficacy and a non-significant small effect size for the long-term efficacy of PM training in older adults. Consistent with the above-mentioned systematic review, PM training regimes such as implementation intention, cognitive training and strategic memory training were found to have good immediate efficacy. Findings from this meta-analysis provided better-quality evidence with a larger statistical power to support the PM training efficacy. The non-significant small effect size for the long-term efficacy of PM training, however, could have occurred due to the limited follow-up data (*n* = 8) and therefore needs to be interpreted with caution. Although two studies demonstrated the maintenance of training gains at follow-up (Kinsella et al., 2009; Schmidt et al., 2001), most of the training gains were not sustained over time. In particular, Kinsella et al. (2009) only revealed training gains in the experimental task but failed to find significant improvements in the self-reported PM measures, whereas Schmidt et al. (2001) demonstrated similar performances of the control and trained groups at follow-up. Given the limited and mixed findings on long-term efficacy, the data need to be interpreted with caution. These findings also indicate a need for continual support after training, which could be provided by booster sessions. Insel et al. (2016) also proposed that integrating these strategies into everyday life might help maintain the long-term gains.

The included studies demonstrated a significantly large immediate efficacy, but not long-term efficacy, for PM training. These discrepancies between immediate and long-term efficacy might be due to a decline in performance over time. A study on implementation intention, one of the most commonly used PM training regimes, also demonstrated a similar effect (Chen et al., 2019); they found a significant improvement in PM at post-test, as measured by the computerised PM task (Ƞp^2^ = 0.17 for EBPM and Ƞp^2^ = 0.11 for TBPM) and the naturalistic PM task (i.e., phone call task; Ƞp^2^ = 0.10). At the three-month follow-up, although the implementation intention group still demonstrated a significant effect in the EBPM measures and phone call task, the overall effect size in the EBPM measures dropped (i.e., Ƞp^2^ from 0.17 to 0.11) and the effect size in the phone call task increased (i.e., Ƞp^2^ from 0.10 to 0.11). These results are similar to those of the current review, which implies that training efficacy fades over time. A case analysis of PM training reported that one of the participants experienced a long-term benefit of implementation intention training, but forgot many elements of the strategy over time (Burkard et al., 2014a). Future studies can conduct booster sessions, which would revise the techniques and help sustain the long-term efficacy. A recent study on a compensatory memory rehabilitation program showed that a booster session after a six-week follow-up assessment could better maintain the training gains (Lawson et al., 2020). Therefore, the lack of significant long-term efficacy in the meta-analysis was possibly due to the small number of studies included. The above evidence indicates that the training gains can be sustained through booster or additional follow-up sessions. This offers a potential area of investigation for future studies on the methods to maintaining training efficacy.

#### Study Populations

We found that both the healthy (*g* = 0.53) and clinical populations (*g* = 0.57) benefited from PM training, albeit possibly through different mechanisms. Previous studies have reported age-related PM decline in healthy older adults (Ball et al., 2019; Gonneaud et al., 2017; Lamichhane et al., 2018), which supports the association between ageing and PM decline. In the included studies, the clinical populations demonstrated a significantly poorer PM performance than the healthy populations (Kinsella et al., 2016; Lee et al., 2016; Mateos et al., 2016; Ozgis et al., 2009; Pereira et al., 2015, 2018; Shelton et al., 2016), which supports a deficit in PM. The positive training effect found for the healthy and clinical older populations may reflect an improvement in PM in the training group compared with the control group. It should be noted that the decline, deficits and improvement reported are based on group averages rather than for all individuals in the groups. The training gains of the two populations showed mixed trends. Some studies proposed that the healthy population stood to gain more than the clinical populations (Kinsella et al., 2016; Pereira et al., 2018), whereas others proposed that the clinical population would have a larger training gain (Lee et al., 2016; Mateos et al., 2016; Ozgis et al., 2009). One study showed no interaction effects between the cognitive status and training conditions (Pereira et al., 2015). Regarding the mixed results in the comparisons of training efficacy between the healthy and clinical populations, it may reflect the phenomenon that both groups benefit from the PM training and cognitive status was not a significant moderator of the training efficacy in the current meta-analysis. However, it is possible that the PM performance of the clinical population will never be restored to that of a normally functioning population. Further studies are required to understand the mechanisms by which PM training exerts its effects in healthy and clinical populations.

It is possible that PM training enhances PM in the healthy population through better monitoring and resource allocation. Most of the training regimes used for this population harness encoding strategies such as implementation intention, enactment encoding, and planning, which mainly target intention formation and retention. For the clinical population, PM training may have improved PM by strengthening key neurocognitive resources such as attentional capacity, processing speed, executive control, metacognition and working memory (Henry, 2021). Unlike the training provided to the healthy population, most of the training regimes provided to the clinical population are comprehensive programme-based interventions such as cognitive training and memory interventions. These interventions support the key neurocognitive resources and thus improve the performance in PM tasks. For instance, cognitive training that targets attention and executive functions might boost any of the phases of PM, such as intention formation, retention, initiation and execution (Poptsi et al., 2017). In contrast, cognitive training involving the deep processing of information, rehearsal and errorless learning consolidates the formation and retention of intention (Tsantali et al., 2017). Likewise, PM training might also remediate PM impairments that are independent of key neurocognitive resources. For example, Tappen and Hain (2013) and Ozgis et al. (2009) showed that spaced retrieval could elevate PM performance. The involvement of a rote process in spaced retrieval implies that strengthening intention formation and retention can alleviate PM impairment in the clinical population.

#### Number of Training Sessions

The number of training sessions did not moderate the training efficacy. Instead, both programme-based (*g* = 0.39) and single-session training (*g* = 0.66) demonstrated positive and robust training efficacies.

Several features of the training regimes may contribute to the significant effect size reported in the programme-based training. First, most of the training programmes targeted cognitive functions such as memory (Emsaki et al., 2017; Kinsella et al., 2009; Schmidt et al., 1999; Troyer et al., 2008), attention, and executive function (Poptsi et al., 2017). Boosting cognitive abilities can improve PM performance. Importantly, PM training that includes metacognitive factors often use everyday experiences, allowing older adults to apply these strategies to their daily lives. Not only does it promote training gains in PM, but it also transfers the training gains to everyday functioning and psychological well-being (Farzin et al., 2018). Likewise, studies that emphasise reflection or generalisation to everyday functioning have revealed positive training gains (Lee et al., 2013; Troyer, 2001; Tsantali et al., 2017; Villa & Abeles, 2000), likely because the application of these strategies to everyday activities consolidates the training gains. Another reason may be the training strategies used in the regimes. Cognitive training (Poptsi et al., 2017; Tappen & Hain, 2013; Tsantali et al., 2017), spaced retrieval, errorless learning (Lee et al., 2013; Tappen & Hain, 2013), and mnemonic strategies (Insel et al., 2016; Kinsella et al., 2009; Schmidt et al., 2001; Troyer et al., 2008; Villa & Abeles, 2000) usually involve multiple practice sessions. These programmes also give feedback to the participants, which help them consolidate the training gains and learn at their own pace.

The meta-analysis revealed that 14 of the 22 single-session training regimes used implementation intention. A previous systematic review and meta-analysis support this finding, having reported a significant and moderate effect of implementation intention in older adults (Cohen’s *d* = 0.68; Chen et al., 2015). Although a single session of implementation intention can effectively improve PM in older adults, the long-term efficacy and generalisability remained unknown. All of the included studies that examined long-term efficacy used programme-based training, which does not clarify the long-term effects of single-session training. To better understand the long-term efficacy of single-session PM training, future studies are suggested to include a follow-up assessment and further examine the maintenance of training gains.

Both programme-based and single-session training regimes can alleviate PM impairment. Programme-based training provides comprehensive training and improves PM performance through learning and practice. Depending on the strategy used, training can improve any of the phases of PM, such as intention formation, retention, initiation and execution. On the contrary, most of the single-session training regimes used implementation intention, enactment encoding, planning, and spaced retrieval, which usually only target intention formation and retention. The healthy population received mostly single-session training (17/25), so we found no conclusive results on the training effect of programme-based training for this population. We only found that single-session training could efficaciously improve age-related PM decline in healthy older adults. In contrast, as most of the clinical population received programme-based training (9/14), it was difficult to conclude whether single-session training also worked for the clinical population. Therefore, from the current analyses, we could only infer that single-session training (e.g. mainly implementation intention and encoding strategies) is efficacious for a healthy older population, while programme-based training (e.g., cognitive training, spaced retrieval and errorless learning) is efficacious for a clinical population. Future studies are suggested to explore the interactions between populations (healthy or clinical population) and the number of training sessions (programme-based or single-session).

### Quality of Evidence

#### Bias and Heterogeneity

The summary risk of bias for the studies included in this review varied widely. Most of the RCTs were rated as having some concern, while most of the non-RCTs were rated as having either a serious or critical risk of bias. The most common reasons for the RCTs to be rated as some concerns or high risk were attrition and the lack of a blinded assessor, which would have resulted in a bias of deviations from the intended interventions and a bias in subjective outcome measures. The participants might have been overly optimistic about the intervention and indicated a larger improvement in the self-reported measures than actually was the case. However, only 22% of the studies were rated as having a high risk of bias, and the sensitivity analyses found no significant differences between the three ratings. To provide a better level of evidence, future studies are suggested to consider including a blinded outcome assessor or adding an objective measure.

For non-RCTs, the risk of bias was mainly serious or critical, which made the evidence of PM training inconclusive. Specifically, most of the studies did not control for confounding variables such as demographics and population groups. Recruiting both healthy and clinical populations concurrently may have confounded the results as the two groups may have had different abilities and performance levels. Future studies should separate the two population groups to more thoroughly investigate the effects of PM training.

Although the qualities of the non-RCT studies were mainly poor, those of the RCT studies were sound. Therefore, the meta-analysis revealed some positive evidence for PM training. More good-quality RCTs are recommended in the future to provide the highest-possible level of evidence.

The heterogeneity between the RCTs in the meta-analysis was moderate-to-high. Although Egger’s test did not show any significant publication bias, a possible modest publication bias was found in the immediate efficacy studies.

### Limitations

There are some limitations to this review. First, the number of included studies that examined the long-term gains of PM training was small. Most of the training, especially the single-session regimes, involved only a post-test time point and did not examine the subsequent long-term gains. Knowing the long-term effects of PM training may help outline a better training approach for follow-up care and arrangement and address the need for continual support after training. Hence, we suggest that future studies include more follow-up sessions and study the long-term gains of PM training.

Second, the generalisation of PM training to everyday functioning remains unclear. As PM is highly related to everyday functioning and psychological well-being, it is important to investigate whether the training gains are transferable to everyday functions. As PM training can enhance PM in both healthy and clinical populations, it might help prevent further cognitive decline by actively engaging the populations in mental exercise. Therefore, future studies should consider extending the findings and studying the generalisation and preventive effects of PM training, which would increase the practical value of PM training.

Third, there were a lot of variations in the PM training approaches and outcome measures. Due to the diversity in training approaches, it is difficult to conclude which training approach is best suited for older adults. Most of the current PM training regimes are strategy-based, and there is insufficient research on process-based training. The variations were also reflected in the meta-analysis, in which we found a significant, moderate-to-high level of heterogeneity (73.97%) in the immediate efficacy of PM training. Although moderator analyses were conducted on the study population and number of training sessions, we could not perform further analysis with the small number of studies examining process-based training (*n* = 3). To better understand the differences and dynamics between strategy-based and process-based training, more studies are required. A large variety of outcome measures was used in the included studies, and very few studies separately examined the training gains in EBPM and TBPM. The different outcome measures used might reflect different levels of training gains. For instance, an objective PM measure (e.g., CAMPROMPT) may demonstrate a more comprehensive and unbiased PM performance than a self-reported PM measure (e.g., PRMQ). In light of these differences, future studies should standardise the training approaches and outcome measures.

Similarly, due to the limited number of studies that examined PM training in different clinical subgroups (e.g., MCI, AD, and PD), subsequent moderator analyses could not be performed. Among the 18 studies involving the clinical population, five studies examined amnestic MCI, four were on MCI or early AD, four investigated mild AD, two were on stroke, one was on severe memory impairment, and two examined mixed diagnosis. Because of the diversity in diagnoses and the small number of studies in each group, we could not examine the efficacy of PM training in each clinical group separately. Future studies should examine this aspect as the deficits in and needs of different clinical populations are likely different.

Due to the small number of included studies that examined the differences in training efficacy between younger and older populations (*n* = 6; Altgassen et al., 2015; Shum et al., 2013; Niedźwieńska et al., 2014; Pereira et al., 2012; Pereira et al., 2018; Zimmermann & Meier, 2010), this review only focused on the PM training efficacy in older adults. We recommend that future reviews assess the differences in training efficacy between younger and older populations.

Finally, the mechanism by which training improves PM remains unclear, regardless of the positive training gains. The corresponding mechanisms underlying the positive findings in both healthy and clinical populations also need to be uncovered. The limited number of studies on process-based training makes it challenging to examine the specific approaches used for PM training (i.e., compensation or restoration). Future studies must focus not only on the outcomes but also on the mechanisms and theoretical basis of PM training. This can be done by conducting neuroimaging experiments to understand the neural mechanism of rehabilitation and by investigating training regimes that target all of the four phases of PM (i.e., intention formation, retention, initiation, and execution).

### Implications

The current review is an update of two previous reviews (Chen et al., 2015; Hering, Cortez, et al., 2014; Hering, Rendell, et al., 2014) and examines the efficacy of PM training more systematically and comprehensively. One of the previous reviews only summarised PM training in older adults and did not assess the efficacy and or consider the different population types (Hering, Rendell, et al., 2014). The other review by Chen et al. (2015) examined the training effects on the healthy and older populations separately, but only focused on implementation intention. Compared with these two previous reviews, the current review confirms the training efficacy of implementation intention and of other PM training approaches such as spaced retrieval, cognitive training, planning and enactment in both healthy and clinical older populations. Furthermore, the findings yield important qualitative and statistical evidence to support PM training, providing a foundation for future theoretical and practical research. In general, regardless of the PM training regime, we found a robust immediate efficacy in both healthy and clinical older populations. More importantly, PM training alleviated the age-related PM decline in the healthy population and the PM deficit in the clinical population. Notwithstanding the diversity in PM training approaches and measured outcomes, the findings from the current review will serve as the latest summary of the efficacy of PM training and help outline a more standardised training approach in future. This review will also help develop strategies that support healthy ageing and address pathological ageing. Our findings also have theoretical implications. It appears that single-session training, which mainly focuses on intention formation and retention, can alleviate PM difficulties in a healthy population, whereas programme-based training, which usually targets all of the four phases of PM, can effectively improve PM deficits in the clinical population. Nevertheless, more studies are needed to verify and support our findings.

## Conclusion

In "Conclusion", this review summarized the currently available PM training regimes and illustrated the immediate efficacy of PM training in both healthy and clinical older adults. Due to the limited number of studies that incorporated follow-up designs and process-based training, the differences in long-term gains and effects between strategy-based and process-based training could not be extensively addressed. Nevertheless, both programme-based and single-session PM training regimes revealed significant training efficacies. Thus, more high-quality studies are needed to enrich this field and provide further evidence to support PM training efficacy in older adults.

**Table 1:** Summary of Interventions (*n* = 58)

	Intervention		
Study Name	Name	Session/ Program	Types of training
Altgassen et al. (2015)	Future thinking	Single session	Strategy-based
Andrewes et al. (1996)	Memory handbook	Program	Strategy-based
Brom and Kliegel (2014)	II	Single session	Strategy-based
Brom et al. (2014)	II	Single session	Strategy-based
Bugg et al. (2013)	II	Single session	Strategy-based
Burkard et al. (2014a)	II intervention (verbal and visual II)	Program	Strategy-based
Burkard et al. (2014b)	II of PM (exclude inhibition)	Single session	Strategy-based
Burkard et al. (2014c)	II	Single session	Strategy-based
Cavallini et al. (2003)	Loci mnemonic & Strategic training	Program	Strategy-based
Chasteen et al. (2001) - Study 1	II - BP task	Single session	Strategy-based
	II - DOW	Single session	Strategy-based
Emsaki et al. (2017)	Memory specificity training	Program	Mixed
Farzin et al. (2018)	PM training program (process and strategy-based component)	Program	Mixed
Foster et al. (2017)	II	Single session	Strategy-based
Goedeken et al. (2018)	II	Single session	Strategy-based
Henry et al. (2021)	II - statement only	Single session	Strategy-based
	II - imagine in game	Single session	Strategy-based
	II - Statement and Imagine combined	Single session	Strategy-based
	II - imagine in daily life	Single session	Strategy-based
Ihle et al. (2018)	Imagery training	Program	Strategy-based
Insel et al. (2016)	Multifaceted PM training (imagine)	Program	Mixed
Kinsella et al. (2009)	Memory intervention	Program	Strategy-based
Kinsella et al. (2016)	Group memory training	Program	Strategy-based
Lee et al. (2013)	Computerized errorless learning-based memory training program (daily life content)	Program	Process-based
	Therapist-led errorless learning program	Program	Process-based
Lee et al. (2016)	II	Single session	Strategy-based

II Implementation intentions, PM prospective memory, BP background-pattern, DOW day of the week, EBPM event-based prospective memory, TBPM time-based prospective memory

**Table 2:** Summary and Moderator Effect Sizes for Immediate Efficacy

Subgroups		Hedges’ *g* (*k*)
Study Populations		
	Healthy population	0.53*** (25)
	Clinical population	0.57*** (14)
Number of sessions in training		
	Program-based	0.39*** (17)
	Single-session	0.66*** (22)

*** *p* ≤ 0.001

### Supplementary information

Below is the link to the electronic supplementary material.

Supplementary file1 (DOCX 60 KB)

## Data Availability

Data are available upon reasonable request from the first authors.

## Appendix A

### An Example Search Strategy in PubMed

((("prospective memory"[Title/Abstract] OR "prospective memories"[Title/Abstract] OR "memory for intentions"[Title/Abstract] OR "prospective remembering"[Title/Abstract] OR "delayed intention"[Title/Abstract] OR "future memory"[Title/Abstract] OR "intentional memory"[Title/Abstract])) AND ("older adult*"[Title/Abstract] OR aged[Title/Abstract] OR ageing[Title/Abstract] OR aging[Title/Abstract] OR elder*[Title/Abstract] OR senior[Title/Abstract] OR "older people"[Title/Abstract] OR "old age*"[Title/Abstract] OR geriatric[Title/Abstract] OR gerontology[Title/Abstract] OR senescence[Title/Abstract] OR senile[Title/Abstract])) AND (train*[Title/Abstract] OR rehabilitation*[Title/Abstract] OR intervention*[Title/Abstract] OR treatment*[Title/Abstract] OR strateg*[Title/Abstract] OR therap*[Title/Abstract] OR remediation[Title/Abstract] OR "cognitive training"[Title/Abstract] OR "memory training"[Title/Abstract] OR "mental training"[Title/Abstract] OR "brain training"[Title/Abstract] OR "cognitive retraining"[Title/Abstract] OR "memory retraining"[Title/Abstract] OR "cognitive rehabilitation"[Title/Abstract] OR "memory rehabilitation"[Title/Abstract] OR "cognitive stimulation"[Title/Abstract] OR "memory stimulation"[Title/Abstract] OR "cognitive exercise"[Title/Abstract] OR "memory exercise"[Title/Abstract] OR "cognitive intervention"[Title/Abstract] OR "memory intervention"[Title/Abstract] OR "cognitive support"[Title/Abstract] OR "cognitive therapy"[Title/Abstract] OR "memory therapy"[Title/Abstract] OR "memory aid"[Title/Abstract] OR "memory group"[Title/Abstract] OR "memory strategy"[Title/Abstract] OR mnemonic[Title/Abstract] OR "memory management"[Title/Abstract] OR "memory strategy"[Title/Abstract]) OR ("Aged"[Mesh] OR "Senior Centers"[Mesh] OR "Aged, 80 and over"[Mesh]) AND ("Health Services for the Aged"[Mesh] OR "Rehabilitation"[Mesh] OR "Therapeutics"[Mesh]) AND ("prospective memory" OR "prospective memories" OR "memory for intentions" OR "prospective remembering" OR "delayed intention" OR "future memory" OR "intentional memory").

Filter activated: 80 and over: 80 + years, Aged: 65 + years, Middle Aged: 45–64 years, Middle Aged + Aged: 45 + years, Adult: 19 + years.

## Appendix E

### Summary Results of Sensitivity Analyses

#### Risk of Bias

Low risk (*n* = 13): *g* = 0.43, *SE* = 0.17, 95% CI [0.09, 0.77], *p* = 0.012.

Some concerns (*n* = 19): *g* = 0.63, *SE* = 0.14, 95% CI [0.36, 0.91], *p* < 0.001.

High risk (*n* = 7): *g* = 0.51, *SE* = 0.23, 95% CI [0.06, 0.96], *p* = 0.025.

#### Study Designs

Cross-sectional (*n* = 20): *g* = 0.70, *SE* = 0.14, 95% CI [0.43, 0.97], *p* < 0.001.

Longitudinal (*n* = 2): *g* = 0.42, *SE* = 0.38, 95% CI [-0.33, 1.17], *p* = 0.273.

Cross-sectional and longitudinal (*n* = 17): *g* = 0.39, *SE* = 0.15, 95% CI [0.09, 0.68], *p* = 0.010.

#### Pre-Post Correlation

*r* = 0.00.

Immediate efficacy: *g* = 0.50, *SE* = 0.09, 95% CI [0.32, 0.67], *p* < 0.001.

Long-term efficacy: *g* = 0.11, *SE* = 0.12, 95% CI [-0.12, 0.34], *p* = 0.331.

*r* = 0.60.

Immediate efficacy: *g* = 0.54, *SE* = 0.10, 95% CI [0.36, 0.73], *p* < 0.001.

Long-term efficacy: *g* = 0.20, *SE* = 0.14, 95% CI [-0.08, 0.47], *p* = 0.165.

*r* = 0.90.

Immediate efficacy: *g* = 0.63, *SE* = 0.10, 95% CI [0.43, 0.83], *p* < 0.001.

Long-term efficacy: *g* = 0.41, *SE* = 0.19, 95% CI [0.04, 0.78], *p* = 0.032.

## Footnotes

1 In the study by Cavallini et al. (2003), although two older populations (i.e., young-old and old-old) were studied, the ages of the older adults were not related to the aims of this review. Therefore, the effect sizes from the young-old and old-old adults were pooled.

2 In the study by Cavallini et al. (2003), the results of the two interventions (i.e., loci mnemonic and strategic training) were combined and no separate data were provided. However, the study was excluded from the meta-analysis as it provided insufficient quantitative data.

3 Two studies did not provide demographics about age (Andrewes et al., 1996; Ozgis et al., 2009; *N* = 110), while four studies did not report the *SD*s of age (Burkard et al., 2014a; Cavallini et al., 2003; Chasteen et al., 2001; Rose et al., 2015; *N* = 175). Therefore, weighted means and *SD*s of 2,672 participants were reported.

4 Twelve studies reported the demographics of the overall population (Altgassen et al., 2015; Kinsella et al., 2016; Lee et al., 2013; McDougall, 2000; McFarland & Glisky, 2011; Niedźwieńska et al., 2014; Pereira et al., 2012; Pereira et al., 2015; Pereira et al., 2018; Schnitzspahn & Kliegel, 2009; Villa & Abeles, 2000; Zimmermann & Meier, 2010).

5 Eighteen studies did not provide sufficient statistics for discriminating the participants into training and control groups.

6 All of the time units were converted to months by assuming 4 weeks equal 1 month.

7 Farzin et al. (2018) followed up with the participants twice after training i.e., at the 4-week and 12-week time points, and both were included in the calculations.

8 Two studies did not provide information on the length of the interventions (Cavallini et al., 2003; Villa & Abeles, 2000).

9 Andrewes et al. (1996) did not mention the exact number of sessions.

10 Five studies did not provide the frequency of training (Andrewes et al., 1996; Cavallini et al., 2003; Schmidt et al., 1999; Schmidt et al., 2001; Villa & Abeles, 2000).

11 Four studies did not provide the session durations (Andrewes et al., 1996; Ihle et al., 2018; McDaniel et al., 2014; Waldum et al., 2016), and two other studies provided only an approximate range of the session duration (Insel et al., 2016; Villa & Abeles, 2000).

12 In the study by Kinsella et al. (2009), the number of participants were different for the two PM measures, i.e., PM performance of Reminding Task and Envelop Task (*n* = 20) and MMQ-ability (*n* = 22). Twenty-two participants were included as trained participants.
