# Correction to: Prospective Memory Training in Older Adults: A Systematic Review and Meta-Analysis

**DOI:** 10.1007/s11065-022-09565-0

**Published:** 2022-11-08

**Authors:** Zita C. K. Tse, Yuan Cao, James M. Ogilvie, Bolton K. H. Chau, Daphne H. C. Ng, David H. K. Shum

**Affiliations:** 1 Department of Rehabilitation Sciences, The Hong Kong Polytechnic University, Hong Kong, Hong Kong; 2 Research Institute for Smart Ageing, The Hong Kong Polytechnic University, Hong Kong, Hong Kong; 3 Mental Health Research Centre, The Hong Kong Polytechnic University, Hong Kong, Hong Kong; 4 Grififth Criminology Institute, Griffith University, Brisbane, Australia; 5 University Research Facility in Behavioral and Systems Neuroscience, The Hong Kong Polytechnic University, Hong Kong, Hong Kong

**Correction to: Neuropsychology Review**

10.1007/s11065-022-09536-5

In the original published paper, it was belatedly discovered that there was a data entry error in pages 1 and 15. The value of “Hedges’ *g* = 0.21” and “*g* = 0.21” were incorrect and should be corrected to “Hedges’ *g* = 0.20” and “*g* = 0.20”, respectively.

The original article has been corrected.

