# Supplementary material for: Prospective Memory Training in Older Adults: A Systematic Review and Meta-Analysis
Source: Neuropsychol Rev. 2022 May 11;33(2):347–72. doi: 10.1007/s11065-022-09536-5 (PMC10148783; doi:10.1007/s11065-022-09536-5)
Supplement: Supplementary file 1 — Supplementary file1 (DOCX 60 KB) [file 11065_2022_9536_MOESM1_ESM.docx]

**Appendix B**

**Summary of Study Characteristics and Designs for Studies Included in Both Qualitative and Quantitative Reviews (*n* = 48)**

| Study characteristics | | | | | | | | Design | | |
| --- | --- | --- | --- | --- | --- | --- | --- | --- | --- | --- |
| Study | RCT/ non-RCT | Trained *N* | Trained *M*_age_ (*SD*); range | Healthy/ Clinical | Comparison *N* | Comparison *M*_age_ (*SD*); range | Healthy/ Clinical | Study designs | Follow-up | Risk of bias |
| Altgassen et al. (2015) | non-RCT | 20 | Overall sample: 71.93 (5.76); 65-87 | Healthy | 20 | N/A | Healthy | Cross-sectional | N/A | Serious |
| Andrewes et al. (1996) | RCT | 20 | N/A | Healthy | 20 | N/A | Healthy | Cross-sectional and longitudinal | 4-month | Some concerns |
| Brom et al. (2014) | RCT | 19 | 68.53 (5.41); 61-79 | Healthy | 20 | 68.64 (4.69); 61-79 | Healthy | Cross-sectional | N/A | Some concerns |
| Brom & Kliegel (2014) | RCT | 15 | 69.8 (6.3); 60-86 | Healthy | 16 | 68.07 (5.89); 60-86 | Healthy | Cross-sectional | N/A | Some concerns |
| Bugg et al. (2013) | RCT | 24 | 72.96 (5.65); 60-85 | Healthy | 23 | 69.74 (1.09); 60-85 | Healthy | Cross-sectional | N/A | Some concerns |
| Burkard et al. (2014a) | non-RCT | 12 | Overall: 70.3; 60+ | Mixed | N.A. | | | Longitudinal | 5-week | Serious |
| Burkard et al. (2014b) | RCT | 40 | 71.4 (8.5); 60+ | Healthy | 45 | 70 (6.2); 60+ | Healthy | Cross-sectional | N/A | Some concerns |

Appendix B (*continued*)

| Study characteristics | | | | | | | | Design | | |
| --- | --- | --- | --- | --- | --- | --- | --- | --- | --- | --- |
| Study | RCT/ non-RCT | Trained *N* | Trained *M*_age_ (*SD*); range | Healthy/ Clinical | Comparison *N* | Comparison *M*_age_ (*SD*); range | Healthy/ Clinical | Study designs | Follow-up | Risk of bias |
| Burkard et al. (2014c) | RCT | 20 | 77.9 (8.08); 60-89 | Mixed | 24 | 78.5 (7.13); 60-89 | Mixed | Cross-sectional | N/A | Low |
| Cavallini et al. (2003) | RCT | 20 | Overall: 69.3; 60-80 | Healthy | 20 | N.A. | Healthy | Longitudinal | N/A | High |
| Chasteen et al. (2001) - Study 1 | non-RCT | 68 | Overall: 71.35 | Healthy | N.A. | | | Cross-sectional | N/A | Serious |
| Emsaki, NeshatDoost, Tavakoli, and Barekatain et al. (2017) | RCT | 10 | 63.1 (5.56) | aMCI | 10 | 63.7 (7.14) | aMCI | Cross-sectional and longitudinal | 3-month | Some concerns |
| Farzin, Ibrahim, Madon, and Basri et al. (2018) | RCT | 13 | 63.69 (4.83); 55-74 | Healthy | 12 | 62.92 (4.14); 55-71 | Healthy | Cross-sectional and longitudinal | 4-week and 12-week | Low |
| Foster, McDaniel, and Rendell et al. (2017) | RCT | 31 | 62.5 (5.7); 50+ | PD | 31 | 62.7 (5.4); 50+ | PD | Cross-sectional and longitudinal | N/A | Low |

Appendix B (*continued*)

| Study characteristics | | | | | | | | Design | | |
| --- | --- | --- | --- | --- | --- | --- | --- | --- | --- | --- |
| Study | RCT/ non-RCT | Trained *N* | Trained *M*_age_ (*SD*); range | Healthy/ Clinical | Comparison *N* | Comparison *M*_age_ (*SD*); range | Healthy/ Clinical | Study designs | Follow-up | Risk of bias |
| Goedeken, Potempa, Prager, and Foster et al. (2018) | RCT | 25 | 63.8 (4.6); 50+ | PD | 27 | 62.7 (5.5); 50+ | PD | Cross-sectional and longitudinal | N/A | Low |
| Henry et al. (2020) | RCT | TG_1_: 25  TG_2_: 25  TG_3_: 25  TG_4_: 25 | TG_1_: 74.24 (5.52); 65-85  TG_2_: 73.16 (5.19); 65-85  TG_3_: 71.76 (4.33); 65-85  TG_4_: 75.84 (5.89); 65-85 | Healthy | 25 | 74 (6.33); 65-85 | Healthy | Cross-sectional | N/A | Some concerns |
| Ihle et al. (2018) | RCT | 21 | 67.62 (3.58); 62-76 | Healthy | 23 | 68.22 (3.44); 62-76 | Healthy | Cross-sectional and longitudinal | N/A | Low |
| Insel et al. (2016) | RCT | 63 | 76.8 (7.4); 65-91 | Healthy | 59 | 77.2 (7.6); 65-94 | Healthy | Cross-sectional and longitudinal | 5-month | Low |
| Kinsella et al. (2009) | RCT | 22 | 78.86 (5.69) | aMCI | 22 | 74.73 (6.1) | aMCI | Cross-sectional and longitudinal | 4-month | Some concerns |

Appendix B (*continued*)

| Study characteristics | | | | | | | | Design | | |
| --- | --- | --- | --- | --- | --- | --- | --- | --- | --- | --- |
| Study | RCT/ non-RCT | Trained *N* | Trained *M*_age_ (*SD*); range | Healthy/ Clinical | Comparison *N* | Comparison *M*_age_ (*SD*); range | Healthy/ Clinical | Study designs | Follow-up | Risk of bias |
| Kinsella et al., (2016) | RCT | Overall HOA: 113  Overall Clinical: 106 | Overall HOA: 72.27 (6.95); 60+  Overall Clinical: 76.08 (7.1); 60+ | HOA: Healthy  Clinical: aMCI | N.A. | | | Cross-sectional and longitudinal | six-month | High |
| Lee et al. (2013) | RCT | TG_1_: 7  TG_2_: 6 | Overall: 77.7 (6.07); 68-91 | Early AD | 6 | N.A. | Early AD | Cross-sectional and longitudinal | 3-month | Low |
| Lee et al. (2016) | RCT | HOA: 17  Clinical: 17 | HOA: 73.2 (6.2); 65-85  Clinical: 80.5 (5.7); 65-85 | HOA: Healthy  Clinical: Mild AD | HOA: 17  Clinical: 19 | HOA: 74.8 (6.5); 65-85  Clinical: 78.6 (6.4); 65-85 | HOA: Healthy  Clinical: Mild AD | Cross-sectional | N/A | High |
| Liu & Park, (2004) | RCT | 10 | 70.1 (5.1); 60-81 | Healthy | CG_1_:10  CG_2_:11 | CG_1_: 71.2 (6.1); 60-81  CG_2_: 72.1 (4.8); 60-81 | Healthy | Cross-sectional | N/A | Low |

Appendix B (*continued*)

| Study characteristics | | | | | | | | Design | | |
| --- | --- | --- | --- | --- | --- | --- | --- | --- | --- | --- |
| Study | RCT/ non-RCT | Trained *N* | Trained *M*_age_ (*SD*); range | Healthy/ Clinical | Comparison *N* | Comparison *M*_age_ (*SD*); range | Healthy/ Clinical | Study designs | Follow-up | Risk of bias |
| Mateos et al. (2016) | non-RCT | TG_1_: 73;  TG_2_: 72 | TG_1_: 75.32 (6.32); 60+;  TG_2_: 74.8 (6.39); 60+ | TG_1_: Clinical: Severe memory impairment;  TG_2_: Healthy | N.A. | | | Longitudinal | N/A | Moderate |
| McDaniel et al. (2014) | RCT | 23 | 64 (4); 55-75 | Healthy | 25 | 64 (7); 55-75 | Healthy | Cross-sectional and longitudinal | N/A | Some concerns |
| McDougall (2000) | non-RCT | 16 | Overall: 83.47 (8.2) | Healthy | 3 | N.A. | Healthy | Cross-sectional and longitudinal | N/A | Critical |
| McFarland & Glisky (2011) | non-RCT | Overall TG_1_: 16  Overall TG_2_: 16 | Overall TG_1_: 75.38 (5.1); 65+  Overall TG_2_: 77.27 (7.32); 65+ | Healthy | N.A. | | | Cross-sectional | N/A | Low |
| Niedźwieńska et al. (2014) | RCT | TG_1_: 25  TG_2_: 24 | Overall: 70.36 (5.66); 61-87 | Healthy | 23 | N.A. | Healthy | Cross-sectional | N/A | Low |

Appendix B (*continued*)

| Study characteristics | | | | | | | | Design | | |
| --- | --- | --- | --- | --- | --- | --- | --- | --- | --- | --- |
| Study | RCT/ non-RCT | Trained *N* | Trained *M*_age_ (*SD*); range | Healthy/ Clinical | Comparison *N* | Comparison *M*_age_ (*SD*); range | Healthy/ Clinical | Study designs | Follow-up | Risk of bias |
| Ozgis et al. (2009) | RCT | Overall HOA: 40  Overall Clinical: 30 | N.A. | HOA: Healthy  Clinical: MCI | N.A. | | | Cross-sectional | N/A | Some concerns |
| de Souza Pereira & Mansur-Alves (2020) | non-RCT | 11 | 75.82 (8.63); 60-93 | Healthy | 10 | 79 (9.7); 60-93 | Healthy | Cross-sectional and longitudinal | N/A | Moderate |
| Pereira et al (2012) | non-RCT | 64 | Overall: 71.17 (7.20); 58-90 | Healthy | N.A. |  |  | Cross-sectional | N/A | Low |
| Pereira et al. (2015) | non-RCT | Overall HOA: 64  Overall Clinical: 64 | Overall HOA: 69.72 (9.8)  Overall Clinical: 72.97 (8.93) | HOA: Healthy  Clinical: MCI | N.A. | | | Cross-sectional | N/A | Some concerns |
| Pereira et al. (2018) | RCT | Overall HOA: 32  Overall Clinical: 32 | Overall HOA: 76.06 (6.03); 62-84  Overall Clinical: 76.75 (5.88); 64-87 | HOA: Healthy  Clinical: aMCI | N.A. | | | Cross-sectional | N/A | Low |

Appendix B (*continued*)

| Study characteristics | | | | | | | | Design | | |
| --- | --- | --- | --- | --- | --- | --- | --- | --- | --- | --- |
| Study | RCT/ non-RCT | Trained *N* | Trained *M*_age_ (*SD*); range | Healthy/ Clinical | Comparison *N* | Comparison *M*_age_ (*SD*); range | Healthy/ Clinical | Study designs | Follow-up | Risk of bias |
| Poptsi et al. (2017) | RCT | 32 | 75.15 (4.87) | Mild AD | 23 | 73 (7.09) | Mild AD | Cross-sectional and longitudinal | N/A | High |
| Rebok et al. (1997) | RCT | TG_1_: 11  TG_2_: 10 | T_1_: 77.5 (6.4); 65-88  T_2_: 78.1 (8.7); 65-88 | Healthy | 11 | 82.3 (9.3); 65-88 | Healthy | Cross-sectional and longitudinal | N/A | Some concerns |
| Rose et al. (2015) | non-RCT | 23 | 67.4; 61-78 | Healthy | CG_1_:18  CG_2_:14 | CG_1_: 68.5; 60-77  CG_2_: 66.4; 60-79 | Healthy | Cross-sectional and longitudinal | N/A | Moderate |
| Schmidt et al. (1999) | RCT | 23 | 63.2 (10); 45-84 | Healthy | 22 | 6.1 (7.3); 46-74 | Healthy | Cross-sectional and longitudinal | 3-month | Low |
| Schmidt et al. (2001) | RCT | TG_1_: 20  TG_2_: 23 | TG_1_: 61.9 (9.8); 45-81  TG_2_: 63.2 (10.1); 45-84 | Healthy | 22 | 61.1 (7.3); 46-74 | Healthy | Cross-sectional and longitudinal | 3-month | High |
| Schnitzspahn & Kliegel (2009) | RCT | Overall TG_1_: 32  Overall TG_2_: 39 | Overall TG_1_: 68.2 (4.2); 60-75  Overall TG_2_: 81.5 (2.9); 76-90 | Healthy | N.A. | | | Cross-sectional | N/A | Some concerns |

Appendix B (*continued*)

| Study characteristics | | | | | | | | Design | | |
| --- | --- | --- | --- | --- | --- | --- | --- | --- | --- | --- |
| Study | RCT/ non-RCT | Trained *N* | Trained *M*_age_ (*SD*); range | Healthy/ Clinical | Comparison *N* | Comparison *M*_age_ (*SD*); range | Healthy/ Clinical | Study designs | Follow-up | Risk of bias |
| Shelton et al. (2016) | RCT | HOA: 17  Clinical: 14 | HOA: 73.2 (6.2); 65-82  Clinical: 80.5 (6.1); 65-85 | HOA: Healthy  Clinical: Mild AD | HOA: 19  Clinical: 17 | HOA: 74.8 (6.5); 65-82  Clinical: 78.6 (6.4); 65-85 | HOA: Healthy  Clinical: Mild AD | Cross-sectional | N/A | High |
| Shum et al. (2013) | RCT | 20 | 66.7 (4.29); 60-75 | Healthy | 20 | 69.3 (4.28); 60-75 | Healthy | Cross-sectional | N/A | Some concerns |
| Tappen & Hain (2013) | RCT | 37 | 80.94 (5.46) | MCI or early AD | 31 | 81.83 (5.79) | MCI or early AD | Cross-sectional and longitudinal | N/A | Low |
| Troyer et al. (2008) | RCT | 24 | 76 (5.6) | aMCI | 24 | 74.8 (7.7) | aMCI | Cross-sectional and longitudinal | 3-month | Some concerns |
| Troyer (2001) | non-RCT | 36 | 72.6 (9.1) | Healthy | 24 | 71.1 (10) | Healthy | Cross-sectional and longitudinal | N/A | Moderate |
| Tsantali et al. (2017) | RCT | TG_1_: 17  TG_2_: 17 | TG_1_: 73.4 (5.7); 67-82  TG_2_: 73.3 (4.9); 67-82 | Mild AD | 21 | 74.2 (5.6); 67-82 | Mild AD | Cross-sectional and longitudinal | 12-month | High |
| Villa & Abeles (2000) | RCT | 38 | Overall: 67.37 (7.39); 55-81 | Healthy | 30 | N.A. | Healthy | Longitudinal | N/A | Some concerns |

Appendix B (*continued*)

| Study characteristics | | | | | | | | Design | | |
| --- | --- | --- | --- | --- | --- | --- | --- | --- | --- | --- |
| Study | RCT/ non-RCT | Trained *N* | Trained *M*_age_ (*SD*); range | Healthy/ Clinical | Comparison *N* | Comparison *M*_age_ (*SD*); range | Healthy/ Clinical | Study designs | Follow-up | Risk of bias |
| Waldum et al. (2016) | non-RCT | 47 | 64.08 (4.61); 55-75 | Healthy | 15 | 64.4 (4.83); 56-72 | Healthy | Cross-sectional and longitudinal | N/A | Serious |
| Zimmermann & Meier (2010) | RCT | 185 | Overall: 68.7 (3.3); 64-75 | Healthy | N.A. | | | Cross-sectional | N/A | High |
| Zöllig et al. (2012) | RCT | 19 | 74.7 (4.19); 69-83 | Healthy | 20 | 75.7 (4.33); 69-83 | Healthy | Cross-sectional | N/A | Low |

*Note*. RCT = randomized control trail; aMCI = amnestic mild cognitive impairment; MCI = mild cognitive impairment; PD = Parkinson’s disease; AD = Alzheimer’s disease; TG = training group; CG = control group; HOA = healthy older adults.

**Appendix C**

**Summary of Interventions and Outcomes in Both Qualitative and Quantitative Reviews (*n* = 48)**

|  | Intervention | | | | | | Outcomes | | |
| --- | --- | --- | --- | --- | --- | --- | --- | --- | --- |
| Study Name | Name | Session/ Program | Session length (min) | Sessions/ Weeks | Total number of sessions | Types of training | Name | Results | Overall result |
| Altgassen et al. (2015) | Future thinking | Single session | N.A. | N.A. | N.A. | Strategy-based | Dresden Breakfast Task | Improved | Improved |
| Andrewes et al. (1996) | Memory handbook | Program | N.A. | N.A. | N.A. | Strategy-based | Laboratory Prospective memory Assessment | No significant difference | Mixed |
|  |  |  |  |  |  |  | Everyday PM assessment | No significant difference |  |
|  |  |  |  |  |  |  | Memory diary | Improved |  |
| Brom et al. (2014) | II | Single session | N.A. | N.A. | N.A. | Strategy-based | Number of forgotten blood pressure test | Improved | Improved |
| Brom & Kliegel (2014) | II | Single session | N.A. | N.A. | N.A. | Strategy-based | Everyday PM task | Improved | Improved |
| Bugg et al. (2013) | II | Single session | N.A. | N.A. | N.A. | Strategy-based | PM hits in active-PM phase | No significant difference | Mixed |
|  |  |  |  |  |  |  | Commission errors in the finished-PM phase | Impaired |  |

Appendix C (*continued*)

|  | Intervention | | | | | | Outcomes | | |
| --- | --- | --- | --- | --- | --- | --- | --- | --- | --- |
| Study Name | Name | Session/ Program | Session length (min) | Sessions/ Weeks | Total number of sessions | Types of training | Name | Results | Overall result |
| Burkard et al. (2014a) | II intervention (verbal and visual II) | Program | 60 | 2 | 10 | Strategy-based | Semi- ecological EBPM task | No significant difference | Mixed |
|  |  |  |  |  |  |  | 1-item PM | No significant difference |  |
|  |  |  |  |  |  |  | GDBS (total) | Improved |  |
| Burkard et al. (2014b) | II of PM (exclude inhibition) | Single session | N.A. | N.A. | N.A. | Strategy-based | PM: writing day of the week | Improved | Improved |
| Burkard et al. (2014c) | II | Single session | N.A. | N.A. | N.A. | Strategy-based | PM tasks (put card in envelop, write name on envelop, ask for questionnaire copy and write required information) | No significant difference | Not improved |
| Cavallini et al. (2003) | Loci mnemonic & Strategic training | Program | 90 | N.A. | 5 | Strategy-based | Memory for activities planned for the week | Improved | Improved |

Appendix C (*continued*)

|  | Intervention | | | | | | Outcomes | | |
| --- | --- | --- | --- | --- | --- | --- | --- | --- | --- |
| Study Name | Name | Session/ Program | Session length (min) | Sessions/ Weeks | Total number of sessions | Types of training | Name | Results | Overall result |
| Chasteen et al. (2001) - Study 1 | II - BP task | Single session | N.A. | N.A. | N.A. | Strategy-based | BP - accuracy | No significant difference | Not improved |
|  |  |  |  |  |  |  | BP - response time | No significant difference |  |
|  | II - DOW | Single session | N.A. | N.A. | N.A. | Strategy-based | DOW | Improved | Improved |
| Emsaki et al. (2017) | Memory specificity training | Program | 80 | 1 | 5 | Mixed | PRMQ | Impaired | Negative |
| Farzin et al. (2018) | PM training program (process and strategy-based component) | Program | 120 | 1 | 6 | Mixed | VW Board Game (objective PM) - TBPM, EBPM, ABPM | Improved | Improved |
|  |  |  |  |  |  |  | PRMQ (self-reported PM) | Improved |  |

Appendix C (*continued*)

|  | Intervention | | | | | | Outcomes | | |
| --- | --- | --- | --- | --- | --- | --- | --- | --- | --- |
| Study Name | Name | Session/ Program | Session length (min) | Sessions/ Weeks | Total number of sessions | Types of training | Name | Results | Overall result |
| Foster et al. (2017) | II | Single session | N.A. | N.A. | N.A. | Strategy-based | VW Board Game - repeated EBPM | No significant difference | Mixed |
|  |  |  |  |  |  |  | VW Board Game - repeated TBPM | No significant difference |  |
|  |  |  |  |  |  |  | VW Board Game - nonrepeated EBPM | No significant difference |  |
|  |  |  |  |  |  |  | VW Board Game - nonrepeated TBPM | No significant difference |  |
| Goedeken et al. (2018) | II | Single session | N.A. | N.A. | N.A. | Strategy-based | PRMQ-Pro | Sustained PM | Mixed |
| Henry et al. (2020) | II - statement only | Single session | N.A. | N.A. | N.A. | Strategy-based | VW Board Game - irregular EBPM | Improved | Mixed |
|  |  |  |  |  |  |  | VW Board Game - irregular TBPM | No significant difference |  |
|  | II - imagine in game | Single session | N.A. | N.A. | N.A. | Strategy-based | VW Board Game - irregular EBPM | Improved | Mixed |
|  |  |  |  |  |  |  | VW Board Game - irregular TBPM | No significant difference |  |

Appendix C (*continued*)

|  | Intervention | | | | | | Outcomes | | |
| --- | --- | --- | --- | --- | --- | --- | --- | --- | --- |
| Study Name | Name | Session/ Program | Session length (min) | Sessions/ Weeks | Total number of sessions | Types of training | Name | Results | Overall result |
|  | II - Statement and Imagine combined | Single session | N.A. | N.A. | N.A. | Strategy-based | VW Board Game - irregular EBPM | Improved | Mixed |
|  |  |  |  |  |  |  | VW Board Game - irregular TBPM | No significant difference |  |
|  | II - imagine in daily life | Single session | N.A. | N.A. | N.A. | Strategy-based | VW Board Game - irregular EBPM | Improved | Mixed |
|  |  |  |  |  |  |  | VW Board Game - irregular TBPM | No significant difference |  |
| Ihle et al. (2018) | Imagery training | Program | N.A. | 2 | 8 | Strategy-based | Criterion PM task (Lexical decision ongoing task) | Mixed findings | Mixed |
| Insel et al. (2016) | Multifaceted PM training (imagine) | Program | N.A. | 1 | 4 | Mixed | Medical Adherence | Improved | Improved |
| Kinsella et al. (2009) | Memory intervention | Program | 90 | 1 | 5 | Strategy-based | PM index - envelop task; and RBMT (reminding task) | Improved | Mixed |
|  |  |  |  |  |  |  | MMQ ability | No significant difference |  |

Appendix C (*continued*)

|  | Intervention | | | | | | Outcomes | | |
| --- | --- | --- | --- | --- | --- | --- | --- | --- | --- |
| Study Name | Name | Session/ Program | Session length (min) | Sessions/ Weeks | Total number of sessions | Types of training | Name | Results | Overall result |
| Kinsella et al. (2016) | Group memory training | Program | 120 | 1 | 6 | Strategy-based | CAMPROMPT | No significant difference | Mixed |
|  |  |  |  |  |  |  | MMQ ability | Improved |  |
|  |  |  |  |  |  |  | CAMPROMPT | No significant difference |  |
|  |  |  |  |  |  |  | MMQ ability | No significant difference |  |
| Lee et al. (2013) | Computerized errorless learning-based memory training program (daily life content) | Program | 30 | 2 | 12 | Process-based | BAPM | Improved | Improved |
|  | Therapist-led errorless learning program | Program | 30 | 2 | 12 | Process-based | BAPM | No significant difference | Not improved |

Appendix C (*continued*)

|  | Intervention | | | | | | Outcomes | | |
| --- | --- | --- | --- | --- | --- | --- | --- | --- | --- |
| Study Name | Name | Session/ Program | Session length (min) | Sessions/ Weeks | Total number of sessions | Types of training | Name | Results | Overall result |
| Lee et al. (2016) | II | Single session | N.A. | N.A. | N.A. | Strategy-based | Category decision task (Einstein et al., 2005) (focal) (Healthy population) | No significant difference | Not improved |
|  |  |  |  |  |  |  | Category decision task (Einstein et al., 2005) (non-focal) (Healthy population) | No significant difference |  |
|  |  |  |  |  |  |  | Category decision task (Einstein et al., 2005) (focal) (very mild AD) | No significant difference |  |
|  |  |  |  |  |  |  | Category decision task (Einstein et al., 2005) (non-focal) (very mild AD) | No significant difference |  |
| Liu & Park (2004) | II | Single session | N.A. | N.A. | N.A. | Strategy-based | Average proportion of successful blood pressure tests | Improved | Improved |
| Mateos et al. (2016) | Memory training program | Program | 120 | 1 | 5 | Strategy-based | Spanish version of the RBMT | Improved | Improved |

Appendix C (*continued*)

|  | Intervention | | | | | | Outcomes | | |
| --- | --- | --- | --- | --- | --- | --- | --- | --- | --- |
| Study Name | Name | Session/ Program | Session length (min) | Sessions/ Weeks | Total number of sessions | Types of training | Name | Results | Overall result |
| McDaniel et al. (2014) | Cognitive training | Program | N.A. | 3 | 24 | Mixed | VW Board Game - regular | Improved | Improved |
|  |  |  |  |  |  |  | VW Board Game - irregular | Improved |  |
|  |  |  |  |  |  |  | VW Board Game - TBPM | Improved (descriptive) |  |
| McDougall (2000) | Cognitive-Behavioral Model of Everyday Memory | Program | 90 | 2 | 8 | Strategy-based | RMBT (asking for appointment) | Improved | Improved |
|  |  |  |  |  |  |  | RMBT (belonging) | Improved |  |
|  |  |  |  |  |  |  | RMBT (delivering a message) | Improved |  |
| McFarland & Glisky (2011) | II | Single session | N.A. | N.A. | N.A. | Strategy-based | Multiple choice test of general knowledge and trivia | Improved | Improved |
| Niedźwieńska et al. (2014) | Social feedback | Single session | N.A. | N.A. | N.A. | Strategy-based | VW Board Game - regular EBPM | No significant difference | Mixed |
|  |  |  |  |  |  |  | VW Board Game - regular TBPM | No significant difference |  |
|  |  |  |  |  |  |  | VW Board Game - irregular EBPM | Improved |  |
|  |  |  |  |  |  |  | VW Board Game - irregular TBPM | No significant difference |  |

Appendix C (*continued*)

|  | Intervention | | | | | | Outcomes | | |
| --- | --- | --- | --- | --- | --- | --- | --- | --- | --- |
| Study Name | Name | Session/ Program | Session length (min) | Sessions/ Weeks | Total number of sessions | Types of training | Name | Results | Overall result |
|  | Non-social feedback | Single session | N.A. | N.A. | N.A. | Strategy-based | VW Board Game - regular EBPM | No significant difference | Mixed |
|  |  |  |  |  |  |  | VW Board Game - regular TBPM | Impaired |  |
|  |  |  |  |  |  |  | VW Board Game - irregular EBPM | No significant difference |  |
|  |  |  |  |  |  |  | VW Board Game - irregular TBPM | No significant difference |  |
| Ozgis et al. (2009) | Space retrieval | Single session | N.A. | N.A. | N.A. | Strategy-based | VW Board Game | Improved (descriptive) | Improved |
| de Souza Pereira & Mansur-Alves (2020) | Episodic memory training | Program | 50 | 1 | 5 | Strategy-based | PM subset in NEUPSILIN | No significant difference | Not improved |
| Pereira et al. (2012) | Enactment | Single session | N.A. | N.A. | N.A. | Strategy-based | Proportion of PM cues respond to correctly | Improved | Improved |
| Pereira et al. (2015) | Enactment | single session | N.A. | N.A. | N.A. | Strategy-based | Computerized PM task (nature/ manmade) | Improved | Improved |

Appendix C (*continued*)

|  | Intervention | | | | | | Outcomes | | |
| --- | --- | --- | --- | --- | --- | --- | --- | --- | --- |
| Study Name | Name | Session/ Program | Session length (min) | Sessions/ Weeks | Total number of sessions | Types of training | Name | Results | Overall result |
| Pereira et al. (2018) | Enactment | Single session | N.A. | N.A. | N.A. | Strategy-based | Computer based PM task (Healthy population) | Improved | Improved |
|  |  |  |  |  |  |  | Computer based PM task (aMCI) | Improved |  |
| Poptsi et al. (2017) | Executive function training | Program | 120 | 2 | 80 | Mixed | PM subscale in functional cognitive assessment scale (FUCAS) | Improved (descriptive) | Mixed |
| Rebok et al. (1997) | Memory Power | Program | 45 | 2 | 16 | Strategy-based | HopPro Phone Task | No significant difference | Not improved |
|  | Mega memory | Program | 45 | 2 | 16 | Strategy-based | HopPro Phone Task | No significant difference | Not improved |
| Rose et al. (2015) | 24 levels of VW game | Program | 60 | 3 | 12 | Process-based | VW Board Game | Improved | Mixed |
|  |  |  |  |  |  |  | Call-back task | Improved |  |
|  |  |  |  |  |  |  | N-back and PM cues | No significant difference |  |
|  |  |  |  |  |  |  | Breakfast Task | No significant difference |  |
|  |  |  |  |  |  |  | PRMQ | No significant difference |  |

Appendix C (*continued*)

|  | Intervention | | | | | | Outcomes | | |
| --- | --- | --- | --- | --- | --- | --- | --- | --- | --- |
| Study Name | Name | Session/ Program | Session length (min) | Sessions/ Weeks | Total number of sessions | Types of training | Name | Results | Overall result |
| Schmidt et al. (1999) | Intervention directed at reducing negative stereotypes and worries about memory | Program | 60 | N.A. | 6 | Strategy-based | Intention sum score (Telephone task + Prospective categorization task) | Improved (descriptive) | Mixed |
| Schmidt et al. (2001) | Prospective memory training | Program | 60 | N.A. | 6 | Strategy-based | Category decision task | No significant difference | Mixed |
|  |  |  |  |  |  |  | Telephone task | No significant difference |  |
|  |  |  |  |  |  |  | Subjective evaluation consisted of counting forgotten prospective actions | Improved (descriptive) |  |
| Schnitzspahn & Kliegel (2009) | II (EBPM) | Single session | N.A. | N.A. | N.A. | Strategy-based | Performance in writing day of the week | No significant difference | Not improved |
|  | II (TBPM) | Single session | N.A. | N.A. | N.A. | Strategy-based | Underline word every 2 mins | No significant difference | Not improved |
| Shelton et al. (2016) | II | Single session | N.A. | N.A. | N.A. | Strategy-based | VW Board Game - missed PM responses | Improved | Improved |

Appendix C (*continued*)

|  | Intervention | | | | | | Outcomes | | |
| --- | --- | --- | --- | --- | --- | --- | --- | --- | --- |
| Study Name | Name | Session/ Program | Session length (min) | Sessions/ Weeks | Total number of sessions | Types of training | Name | Results | Overall result |
| Shum et al. (2013) | Planning | Single session | N.A. | N.A. | N.A. | Strategy-based | Performance in home-like laborartory and finding recipes (EBPM) | Improved (descriptive) | Improved |
|  |  |  |  |  |  |  | Performance in home-like laborartory and finding recipes (TBPM) | Improved (descriptive) |  |
|  |  |  |  |  |  |  | Performance in home-like laborartory and finding recipes (ABPM) | Improved (descriptive) |  |
| Tappen & Hain (2013) | In-home cognitive training | Program | 60 | 2 | 24 | Strategy-based | Event-related prospective memory tasks (cash from envelop) | Improved | Mixed |
|  |  |  |  |  |  |  | Event-related prospective memory tasks (water from refrigerator) | No significant difference |  |
| Troyer et al. (2008) | Multidisciplinary group-based intervention program | Program | 120 | 1 | 10 | Strategy-based | MMQ ability | No significant difference | Not improved |
| Troyer (2001) | Memory and aging program | Program | 120 | 1 | 5 | Strategy-based | Telephone call task | Improved | Improved |
|  |  |  |  |  |  |  | MMQ ability | Improved |  |

Appendix C (*continued*)

|  | Intervention | | | | | | Outcomes | | |
| --- | --- | --- | --- | --- | --- | --- | --- | --- | --- |
| Study Name | Name | Session/ Program | Session length (min) | Sessions/ Weeks | Total number of sessions | Types of training | Name | Results | Overall result |
| Tappen & Hain (2013) | In-home cognitive training | program | 60 | 2 | 24 | Strategy-based | Event-related prospective memory tasks (cash from envelop) | Improved | Mixed |
|  |  |  |  |  |  |  | Event-related prospective memory tasks (water from refrigerator) | No significant difference |  |
| Troyer et al. (2008) | Multidisciplinary group-based intervention program | Program | 120 | 1 | 10 | Strategy-based | MMQ ability | No significant difference | Not improved |
| Troyer (2001) | Memory and aging program | Program | 120 | 1 | 5 | Strategy-based | Telephone call task | Improved | Improved |
|  |  |  |  |  |  |  | MMQ ability | Improved |  |
| Tsantali et al. (2017) | Cognitive training | Program | 90 | 3 | 48 | Strategy-based | RBMT (Date/appointment) | Improved | Improved |
|  | Cognitive stimulation | Program | 90 | 3 | 48 | Strategy-based | RBMT (Date/appointment) | No significant difference | Not improved |

Appendix C (*continued*)

|  | Intervention | | | | | | Outcomes | | |
| --- | --- | --- | --- | --- | --- | --- | --- | --- | --- |
| Study Name | Name | Session/ Program | Session length (min) | Sessions/ Weeks | Total number of sessions | Types of training | Name | Results | Overall result |
| Villa & Abeles (2000) | Memory enhancement workshop (laboratory stimuli) | Program | N.A. | N.A. | 7 | Strategy-based | 6 items from PROMS and 3 items from RBMT | Improved | Improved |
|  | Memory enhancement workshop (ecological stimuli) | Program | N.A. | N.A. | 7 | Strategy-based | 6 items from PROMS and 3 items from RBMT | Improved | Improved |
| Waldum et al. (2016) | PM training | Program | N.A. | 1 | 8 | Mixed | TBPM (Famous Faces, Driving Task) | Improved | Mixed |
|  |  |  |  |  |  |  | EBPM | No significant difference |  |
| Zimmermann & Meier (2010) | II | Single session | N.A. | N.A. | N.A. | Strategy-based | Lexical decision task with PM (correct responses) | Improved | Improved |
|  |  |  |  |  |  |  | Lexical decision task with PM (prospective component) | Improved |  |

Appendix C (*continued*)

|  | Intervention | | | | | | Outcomes | | |
| --- | --- | --- | --- | --- | --- | --- | --- | --- | --- |
| Study Name | Name | Session/ Program | Session length (min) | Sessions/ Weeks | Total number of sessions | Types of training | Name | Results | Overall result |
| Zöllig et al. (2012) | Familiarization intervention | Single session | N.A. | N.A. | N.A. | Strategy-based | 2-back working memory task with PM (correct) | No significant difference | Mixed |
|  |  |  |  |  |  |  | 2-back working memory task with PM (error) | No significant difference |  |
|  |  |  |  |  |  |  | 2-back working memory task with PM (false alarm) | Improved |  |

*Note*. II = implementation intentions; PM = prospective memory; EBPM = event-based prospective memory; GDBS = Goal Directed Behavioral Scale; BP = background-pattern; DOW = day of the week; PRMQ = prospective and retrospective memory questionnaire; TBPM = time-based prospective memory; ABPM = activity-based prospective memory; VW = virtual week; RBMT = Rivermead Behavioural Memory Test; MMQ = multifactorial memory questionnaire; CAMPROMPT = Cambridge Test of Prospective Memory; BAPM = Brief Assessment of Prospective Memory; NEUPSILIN = Brief Neuropsychological Assessment Instrument; aMCI = amnestic mild cognitive impairment; FUCAS = Functional Cognitive Assessment Scale; HopPro = Hopkins Prospective Memory Test; PROMS = Prospective Memory Screening Test.

**Appendix D**

**Summary of Overall Effect Sizes (Hedges’ *g*) and Sample Sizes for Meta-Analysis (*n* = 29)**

| Study | Immediate efficacy | | Long-term efficacy | |
| --- | --- | --- | --- | --- |
|  | Hedges’ *g* | *N*  Training (Control) | Hedges’ *g* | *N*  Training (Control) |
| Brom et al. (2014) | 0.82 | 15 (16) | - | - |
| Brom & Kliegel (2014) | 1.22 | 19 (20) | - | - |
| Bugg et al. (2013) | -0.49 | 24 (23) | - | - |
| Burkard et al. (2014b) | 0.89 | 40 (45) | - | - |
| Emsaki et al. (2017) | -1.10 | 9 (8) | -1.16 | 9 (7) |
| Farzin et al. (2018) | 2.32 | 13 (12) | - | - |
| Foster et al. (2017) | 0.15 | 31 (31) | - | - |
| Goedeken et al. (2018) | 0.60 | 25 (27) | - | - |
| Henry et al. (2020) - II imagine in game | 0.22 | 25 (25) | - | - |
| Henry et al. (2020) - II statement and imagine combined | 0.71 | 25 (25) | - | - |
| Henry et al. (2020) - II statement only | 0.29 | 25 (25) | - | - |
| Henry et al. (2020) - imagine in daily life | 0.01 | 25 (25) | - | - |
| Ihle et al. (2018) | -0.95 | 21 (23) | - | - |
| Insel et al. (2016) | 0.78 | 58 (57) | 0.30 | 58 (57) |
| Kinsella et al. (2009) | 0.38 | 21 (21) | 0.31 | 21 (21) |
| Lee et al. (2013) - CELP | 0.60 | 7 (6) | 0.42 | 6 (6) |
| Lee et al. (2013) - TELP | 0.46 | 6 (6) | 0.94 | 7 (6) |
| Lee et al. (2016) - HOA | -0.22 | 17 (19) | - | - |
| Lee et al. (2016) - very mild AD | 0.61 | 11 (12) | - | - |
| Liu & Park (2004) | 0.89 | 10 (11) | - | - |
| McDaniel et al. (2014) - cognitive training only | 0.42 | 18 (19) | - | - |
| Niedźwieńska et al. (2014) - non-social feedback | 0.34 | 24 (25) | - | - |
| Niedźwieńska et al. (2014) - social feedback | 0.46 | 23 (25) | - | - |

Appendix D (*continued*)

| Study | Immediate efficacy | | | Long-term efficacy | |
| --- | --- | --- | --- | --- | --- |
|  | Hedges’ *g* | *N*  Training (Control) | | Hedges’ *g* | *N*  Training (Control) |
| Ozgis et al. (2009) - HOA | 1.42 | | 20 (20) | - | - |
| Ozgis et al. (2009) - MCI | 2.75 | | 15 (15) | - | - |
| Pereira et al. (2018) - aMCI | 1.27 | | 16 (16) | - | - |
| Pereira et al. (2018) - HOA | 1.17 | | 16 (16) | - | - |
| Poptsi et al. (2017) | 1.11 | | 32 (23) | - | - |
| Schmidt et al. (1999) | 0.10 | | 23 (22) | 0.43 | 23 (22) |
| Schmidt et al. (2001) | 0.35 | | 20 (45) | 0.04 | 20 (45) |
| Shum et al. (2013) | 1.86 | | 18 (16) | - | - |
| Tappen & Hain (2013) | 0.28 | | 37 (31) | - | - |
| Troyer et al. (2008) | 0.21 | | 24 (24) | 0.06 | 23 (22) |
| Tsantali et al. (2017) - cognitive stimulation | -0.37 | 17 (21) | | - | - |
| Tsantali et al. (2017) - cognitive training | 1.66 | 17 (21) | | - | - |
| Villa & Abeles (2000) - ecological (longitudinal) | 0.56 | 38 | | - | - |
| Villa & Abeles (2000) - laboratory (longitudinal) | 0.28 | 30 | | - | - |
| Zimmermann & Meier (2010) | 0.50 | 46 (68) | | - | - |
| Zoliget et al. (2012) | 0.59 | 19 (20) | | - | - |

*Note*. II = implementation intentions; CELP = computerized errorless learning-based memory training program; TELP = therapist-led errorless learning program; HOA = healthy older adults; AD = Alzheimer’s disease; MCI = mild cognitive impairment; aMCI = amnestic mild cognitive impairment.
